# Immunoinformatics-aided design of a new multi-epitope vaccine adjuvanted with domain 4 of pneumolysin against *Streptococcus pneumoniae* strains

**DOI:** 10.1186/s12859-023-05175-6

**Published:** 2023-02-24

**Authors:** Mona Shafaghi, Zohreh Bahadori, Hamid Madanchi, Mohammad Mehdi Ranjbar, Ali Akbar Shabani, Seyed Fazlollah Mousavi

**Affiliations:** 1 Department of Medical Biotechnology, Faculty of Medicine, Semnan University of Medical Sciences, Semnan, Iran; 2 Research Center of Biotechnology, Semnan University of Medical Sciences, Semnan, Iran; 3 Department of Bacteriology, Pasteur Institute of Iran, Tehran, Iran; 4 Drug Design and Bioinformatics Unit, Department of Medical Biotechnology, Biotechnology Research Center, Pasteur Institute of Iran, Tehran, Iran; 5 Agricultural Research, Education, and Extension Organization (AREEO), Razi Vaccine and Serum Research Institute, Karaj, Iran

**Keywords:** Immunoinformatics, Multi-epitope vaccine, Pneumococcal surface protein A (PspA), Pneumococcal histidine triad protein D (PhtD), Domain 4 of pneumolysin (Ply4), Protein TLR agonist adjuvant

## Abstract

**Background:**

*Streptococcus pneumoniae* (Pneumococcus) has remained a leading cause of fatal infections such as pneumonia, meningitis, and sepsis. Moreover, this pathogen plays a major role in bacterial co-infection in patients with life-threatening respiratory virus diseases such as influenza and COVID-19. High morbidity and mortality in over one million cases, especially in very young children and the elderly, are the main motivations for pneumococcal vaccine development. Due to the limitations of the currently marketed polysaccharide-based vaccines, non-serotype-specific protein-based vaccines have received wide research interest in recent years. One step further is to identify high antigenic regions within multiple highly-conserved proteins in order to develop peptide vaccines that can affect various stages of pneumococcal infection, providing broader serotype coverage and more effective protection. In this study, immunoinformatics tools were used to design an effective multi-epitope vaccine in order to elicit neutralizing antibodies against multiple strains of pneumococcus.

**Results:**

The B- and T-cell epitopes from highly protective antigens PspA (clades 1–5) and PhtD were predicted and immunodominant peptides were linked to each other with proper linkers. The domain 4 of Ply, as a potential TLR4 agonist adjuvant candidate, was attached to the end of the construct to enhance the immunogenicity of the epitope vaccine. The evaluation of the physicochemical and immunological properties showed that the final construct was stable, soluble, antigenic, and non-allergenic. Furthermore, the protein was found to be acidic and hydrophilic in nature. The protein 3D-structure was built and refined, and the Ramachandran plot, ProSA–web, ERRAT, and Verify3D validated the quality of the final model. Molecular docking analysis showed that the designed construct via Ply domain 4 had a strong interaction with TLR4. The structural stability of the docked complex was confirmed by molecular dynamics. Finally, codon optimization was performed for gene expression in *E. coli*, followed by in silico cloning in the pET28a(+) vector.

**Conclusion:**

The computational analysis of the construct showed acceptable results, however, the suggested vaccine needs to be experimentally verified in laboratory to ensure its safety and immunogenicity.

**Supplementary Information:**

The online version contains supplementary material available at 10.1186/s12859-023-05175-6.

## Background

*Streptococcus pneumoniae,* a commensal Gram-positive bacterium, is one of the leading causes of death worldwide, causing a variety of infections such as pneumonia, meningitis, and bacteremia [1, 2]. The high-risk populations for these severe diseases include children under two years old, the elderly above 65 years old, and immunocompromised patients [3, 4]. Furthermore, viral infections such as influenza and coronavirus pose a high risk of developing severe invasive pneumococcal infections, resulting in high mortality rates [5, 6]. The high cost of antibiotic treatment and increasing the antibiotic resistance make vaccination against pneumococcus a particularly attractive intervention [7]. Currently, there are two kinds of pneumococcal vaccines based on a limited number of serotype-specific capsular polysaccharides; the 23-valent pneumococcal polysaccharide vaccine (PPV), which is consisted of 23 different capsular polysaccharides; and the 7-, 10-, or 13-valent pneumococcal conjugate vaccine (PCV), which is composed of the prevalent polysaccharides conjugated to a carrier protein [8, 9]. The PPV provides good coverage but does not protect at-risk groups under the age of two years, while the PCV induces efficient protective immunity in newborns but presents limited coverage and has a high production cost, and also requires several injections [10, 11]. However, the increase in pneumococcal infections caused by non-vaccine serotypes, due to serotype replacement, reduces the effectiveness of this group of vaccines [12, 13]. In response to these problems, it is necessary to design a novel vaccine that is more affordable and provides broad protection across all pneumococcal serotypes [13, 14]. Protein-based pneumococcal vaccines, containing a combination of the conserved proteins common in most or all of the strains, provide a promising alternative to the existing vaccines [14, 15]. In this context, in recent years, various protein factors involved in colonization and virulence have been investigated, and each of them could induce significantly different levels of protection in animal models and humans [16–18].

The pneumococcal surface protein A (PspA) is one of the most studied virulence factors that has been found in all clinical isolates of *S. pneumoniae* [19]. This antigen helps the pneumococcus escape the defense system of the host by interfering with the deposition of complement molecules on the surface of the bacteria and blocking the bactericidal activity of lactoferrin peptides [20, 21]. The PspA protein has three major domains (Additional file 1: Fig. S1); including (i) amino acids 1–288, an alpha helical domain (α-HD) consisting of A, A′ and B regions; (ii) amino acids 289–370, a proline-rich domain (C region); and (iii) amino acids 371–571, a choline-binding domain responsible for surface attachment [22]. The α-HD and C regions in N-terminal of the protein are surface-exposed and can interact with the host immune system [23, 24]. The α-HD is variable and highly immunogenic, and it appears that protection is caused by epitopes in the 100 amino acids at its N-terminus (A region) and ∼ 100 residues at its C-terminus (B region) [24–26]. PspA is classified into six clades composing three families based on the sequence variability at B region designated as the clade-defining region (CDR) [22]. Family 1 consists of two clades (1 and 2), family 2 consists of three clades (3, 4 and 5), and family 3 has only one clade (6) which is found in 0.1–4% of strains [22]. According to different studies, more than 95% of pneumococcal isolates are in family 1 and family 2, and therefore efforts to develop PspA-based vaccines are focused on these two families [27, 28]. The level of cross-reactivity differs between different PspAs based on the degree of sequence similarities so that there is a greater cross-reactivity within the same clade [29]. Since in some studies it has been found that immune responses triggered by two major families of PspA are clade dependent [29–31], it is proposed that high antigenic regions from all their clades, having highest effect on cross-reactivity, should be included in PspA-based subunit vaccines [32]. Furthermore, the regions A and C in the PspA protein possess conserved epitopes which have the impact on cross-reactivity [31, 33].

Another important vaccine candidate is the Pneumococcal histidine triad protein D (PhtD), that belongs to the polyhistidine triad family and is characterized by the presence of five copies of the His triad motif (HxxHxH) [34]. This highly-conserved protein, which is expressed by all pneumococcal strains, inhibits complement deposition and mediates bacterial adherence by zinc binding [16]. The studies have revealed that the C-terminal fragment of PhtD (PhtD-C) is more surface-exposed and hence could be a more immunogenic region than other regions of the protein [35, 36]. The immunization with the truncated derivatives of PhtD-C was found to be more able to induce antibody responses and protective immunity than the immunization with the full length protein [37].

Adjuvants, which are important factors in vaccine development, are used to induce a faster, more efficient, and longer-lasting immune response [38]. Over the past few decades, protein toll-like-receptor (TLR) agonists have been studied as promising vaccine adjuvant candidates [39]. It has been shown that various bacterial protein TLR agonists exhibit adjuvant properties, such as the activation of TLR signaling, the production of pro-inflammatory cytokines, and maturation of antigen presenting cells. The peptide nature of these potential protein adjuvants provides unique properties, including the capability to modify the structure/function as necessary for minimal toxicity and high immunogenicity. Moreover, they can be genetically fused to peptide antigens that ensure the co-delivery of antigen-adjuvant simultaneously to the same cell, resulting to more effective activation of immune system [40]. Many pneumococcal proteins have been proven to be sensed by toll-like receptors, such as pneumolysin (Ply), DnaJ, RrgA pneumococcal pilus type 1 protein, and so on [40–42]. Studies have demonstrated that the C-terminal domain 4 of pneumolysin (Ply4) alone possesses TLR4 agonist activity [43]. Therefore, it can be suggested that Ply4 could be considered a potential vaccine adjuvant candidate, provided that modifications could be made to eliminate its virulence.

Bioinformatics approaches can assist researchers in various biological fields [44, 45]; particularly, for prediction of potentially immunoprotective epitopes to design candidate vaccines [46, 47]. Actually, the immuno-bioinformatics tools represent advantages over conventional vaccinology methods, including faster results and lower costs [48]. The present study is aimed at designing of a new multi-epitope based subunit vaccine to elicit neutralizing antibody responses against *S. pneumoniae* using immunoinformatics approaches. In view of the importance of the proteins PspA from clades 1–5 (PspA1-5) and PhtD, the subunit vaccine containing proper B- and helper T-cell epitopes of these antigens may serve as an efficient vaccine candidate for a wide range of pneumococcal strains. In this study, it has been focused on the sequences of reference strains that are expected to exhibit a greater diversity than the other isolates [22]. The dominant epitopes were predicted and then the selected peptides were fused together using suitable linkers to construct the main core of the multi-epitope vaccine. Since the epitope vaccine may be quickly degraded by peptidases, the TLR agonist Ply4 was attached to the N-terminal of the construct as a potential adjuvant candidate to overcome this weakness.

## Results

**Fig. 1:**
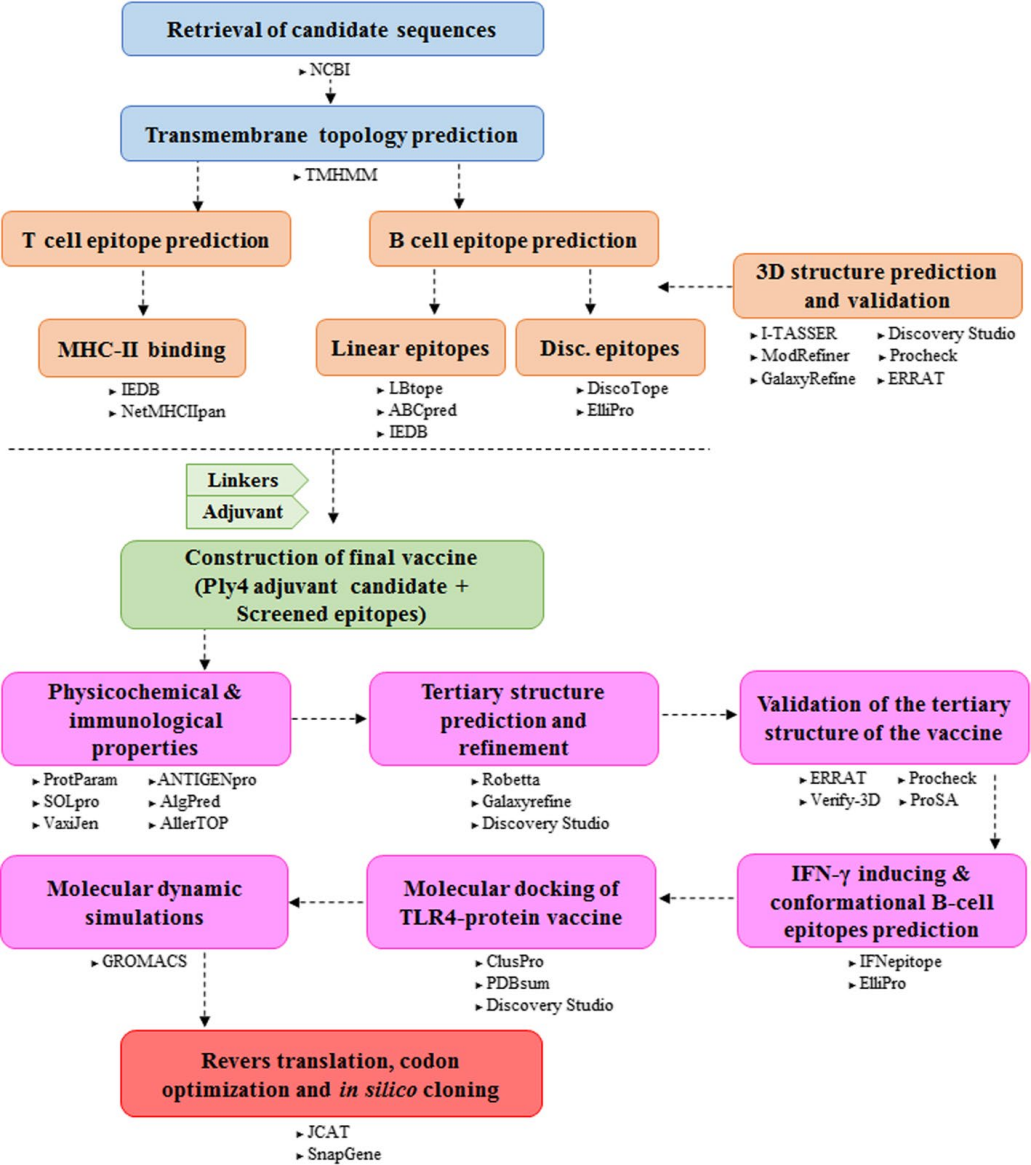
Schematic flowchart of this study for developing the multi-epitope vaccine against *S. pneumoniae*. The entire approach employed in this study are represented in several phases and the used servers/softwares are mentioned below of each phase

The overall workflow utilized in this study for developing the multi-epitope vaccine against *S. pneumoniae* is summarized in Fig. 1.

### Retrieval of the protein sequences

The amino acid sequences of PspA1-5, PhtD and Ply were obtained from GenBank in FASTA format (Additional file 1: Table S1). A region of PspA2 (PspA2-A), B region of PspA1-5 (PspA1-5-B), C region of PspAs (PspA-C) and C-terminal of PhtD (from amino acid 383–853) were selected for B-cell and helper T-cell epitope predictions. Domain 4 of pneumolysin (from amino acid 360–471) was used as a potential TLR4 ligand-based adjuvant to enhance the immunogenicity of the construct. Three mutations Asp385Asn, Cys428Gly and Trp433Phe were done in the domain 4 to eliminate the virulence of pneumolysin.

### Transmembrane domain prediction

The results of the TMHMM server demonstrated that the candidate sequences had no transmembrane domain (Additional file 1: Fig. S2).

### 3D structure prediction and validation of candidate antigens

The 3D structure of A region of PspA2 or PhtD-C was predicted through I-TASSER, and the model No. 1 with the highest score (C-score = − 0.70 or − 0.64, respectively) was refined with ModRefiner and GalaxyRefine (Additional file 1: Fig. S3). The validation results obtained using the SAVES server on the basis of PROCHECK and ERRAT are shown in Additional file 1: Fig. S4. Ramachandran plot of the refined model of PspA2 or PhtD-C indicated 82.1% or 87.2% of amino acids in favored regions, respectively. The ERRAT plot showed that the structure of A region of PspA2 or PhtD-C with overall quality factor 88.69% or 85.23%, respectively, can be acceptable. The tertiary structure modelling of B regions of PspA1-5 was done using Swiss-Model based on the template 2PMS with sequence identity more than 30% (Additional file 1: Fig. S5).

### B cell epitopes prediction

The B cell epitopes were predicted by LBTope, ABCpred, Emini surface accessibility prediction tool of IEDB, Ellipro and DiscoTope servers. The epitopes of A region of PspA2 are listed in Additional file 1: Table S2 and the epitopes of B regions of PspA1-5 are represented in Additional file 1: Table S3–S7, respectively. The B cell epitopes of C region of PspAs were obtained considering only experimentally verified epitopes in recent studies (Additional file 1: Table S8) [23, 24, 49]. The predicted linear and conformational B cell epitopes of PhtD-C are shown in Additional file 1: Table S9 and S10, respectively.

### Prediction of helper T cell (MHC-II) epitopes

The IEDB and NetMHCIIpan 4.0 servers were used to predict the epitopes of MHC class II (8 common human DRB1 alleles: 01:01, 03:01, 04:01, 07:01, 08:01, 11:01, 13:01, and 15:01, as well as 3 mouse alleles: H2-IAb, IAd, and IEd). The epitopes of A region of PspA2, B region of PspA1-5, and PhtD-C with higher binding affinity, based on percentile rank < 10.0 for IEDB and %Rank < 1.0 for NetMHCIIpan SBs, were considered for further analysis. The MHC‑II‑Binding epitopes predicted by IEDB and NetMHCIIpan for PspA (PspA2-A and PspA1-5-B) and PhtD-C are shown in Additional file 1: Table S11 and S12, respectively.

### Final vaccine sequence construction

**Fig. 2:**
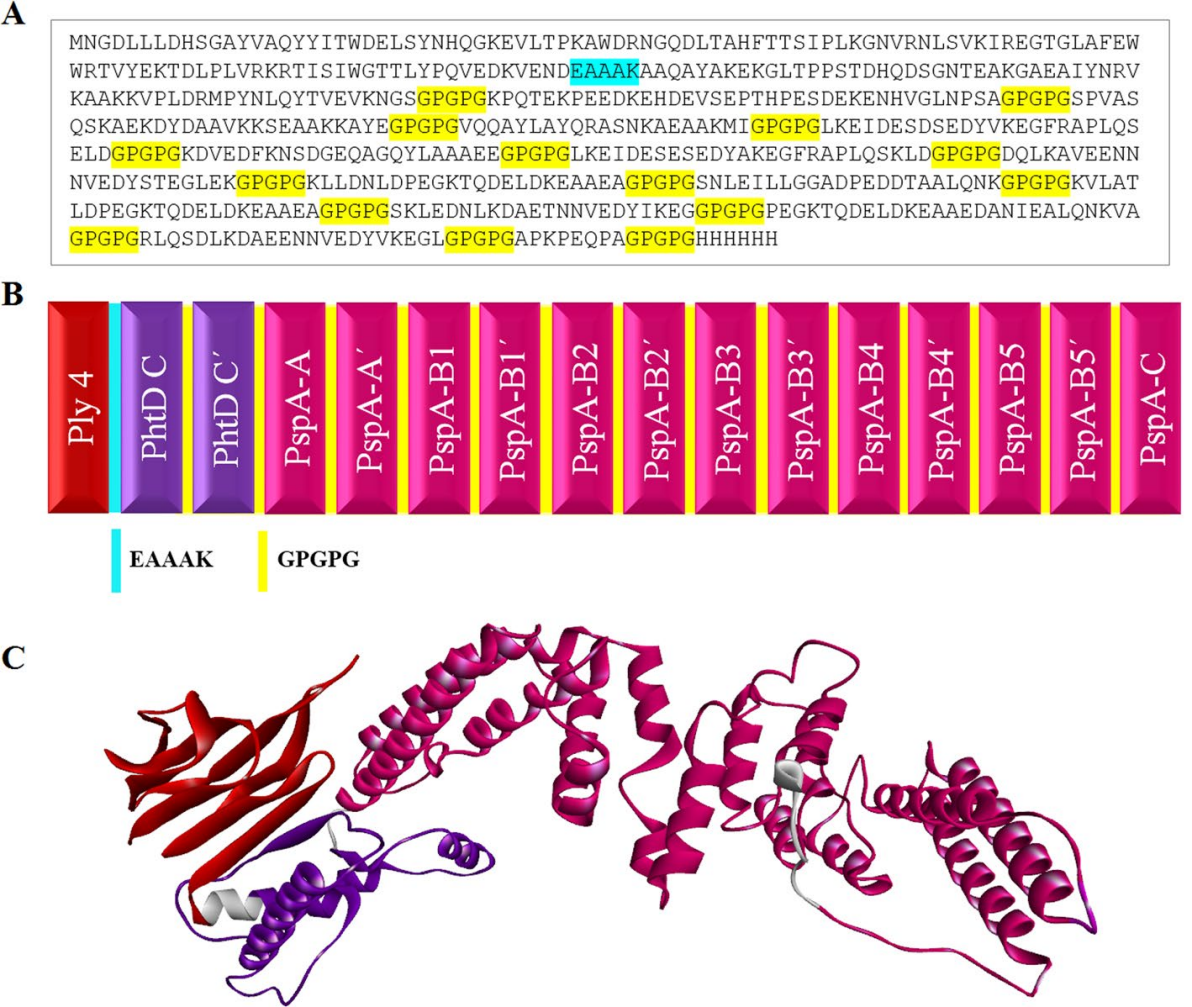
The amino acid sequence, schematic diagram, and 3D refined structure of the vaccine candidate. **A** The final protein sequence of the multi-epitope construct consisted of 590 residues. The linkers EAAAK and GPGPG are shown in cyan and yellow, respectively. **B** The schematic image of vaccine construct was obtained from the selected regions of Ply4, PhtD and PspA, as well as linkers EAAAK and GPGPG, which are shown in red, purple, magenta, cyan and yellow, respectively. **C** The refined model was provided by the GalaxyRefine server and visualized by the Discovery Studio Visualizer. The regions of Ply4, PhtD and PspA are shown in red, purple, and magenta, respectively

Altogether, the high-scored B-cell and MHC-II epitopes shared between different servers were considered, and the 3D structures were used to choose the final suitable domains. The selected peptides of PspA and PhtD for the final construct are listed in Table 1. These peptides were merged together with the help of GPGPG linkers and the overall length of the main vaccine sequence was found to be 461 amino acids. To enhance the immunogenicity of the epitope vaccine, the domain 4 of pneumolysin was considered as an adjuvant candidate. The amino acid sequence of Ply4 containing three mutations D385N, C428G and W433F (112 amino acids long, Table 1) was attached to the N-terminal of the above peptides using an EAAAK linker. A 6xHis-tag was then added by GPGPG linker to the C-terminal of the designed vaccine construct, which could be helpful for the effective identification and purification of the protein. The final vaccine construct with a total of 590 amino acids consisting of the considered peptides of Ply, PhtD and PspA fused together with the suitable linkers is shown in Fig. 2A–B.

**Table 1:** The final selected peptides of PspA and PhtD, and the mutated sequence of domain 4 of Ply included in the vaccine construct

Protein	Region	Peptide sequence	Position
PspA	A Region	SPVASQSKAEKDYDAAVKKSEAAKKAYE	3–30 (PspA2)
		VQQAYLAYQRASNKAEAAKMI	74–94 (PspA2)
	B Region	LKEIDESDSEDYVKEGFRAPLQSELD	182–207 (PspA1)
		KDVEDFKNSDGEQAGQYLAAAEE	266–288 (PspA1)
		LKEIDESESEDYAKEGFRAPLQSKLD	190–215 (PspA2)
		DQLKAVEENNNVEDYSTEGLEK	243–264 (PspA2)
		KLLDNLDPEGKTQDELDKEAAEA	325–347 (PspA3)
		SNLEILLGGADPEDDTAALQNK	368–389 (PspA3)
		KVLATLDPEGKTQDELDKEAAEA	254–276 (PspA4)
		SKLEDNLKDAETNNVEDYIKEG	297–318 (PspA4)
		PEGKTQDELDKEAAEDANIEALQNKVA	258–284 (PspA5)
		RLQSDLKDAEENNVEDYVKEGL	299–320 (PspA5)
	C Region	APKPEQPA	354–361 (PspA1) 426–433 (PspA3) 365–372 (PspA5)
PhtD	C-terminal of PhtD	AAQAYAKEKGLTPPSTDHQDSGNTEAKGAEAIYNRVKAAKKVPLDRMPYNLQYTVEVKNGS	559–619
		KPQTEKPEEDKEHDEVSEPTHPESDEKENHVGLNPSA	700–736
Ply	Domain 4	NGDLLLDHSGAYVAQYYITWDELSYNHQGKEVLTPKAWDRNGQDLTAHFTTSIPLKGNVRNLSVKIREGTGLAFEWWRTVYEKTDLPLVRKRTISIWGTTLYPQVEDKVEND	360–471

### Evaluation of the various features of the designed vaccine

The Expasy ProtParam results revealed that the molecular weight and theoretical pI value of the designed construct were 62.96 kDa and 4.60, respectively. The half-life was computed to be > 10 h in *E. coli*, > 20 h in yeast, and 30 h in mammalian reticulocytes. The instability index, aliphatic index and grand average of hydropathicity were predicted as 33.71, 59.63 and − 1.028, respectively. The protein solubility upon overexpression in *E. coli* was estimated 0.958393 by the SOLpro server, which indicated the protein construct was soluble. The antigenicity of the final construct was checked using the VaxiJen and ANTIGENpro servers to be 0.8833 and 0.850188, respectively. The prediction of allergenicity by AlgPred and AllerTOP servers demonstrated that the multi-epitope vaccine was a non-allergen.

### Prediction, refinement, and validation of the 3D structure of the candidate vaccine

**Fig. 3:**
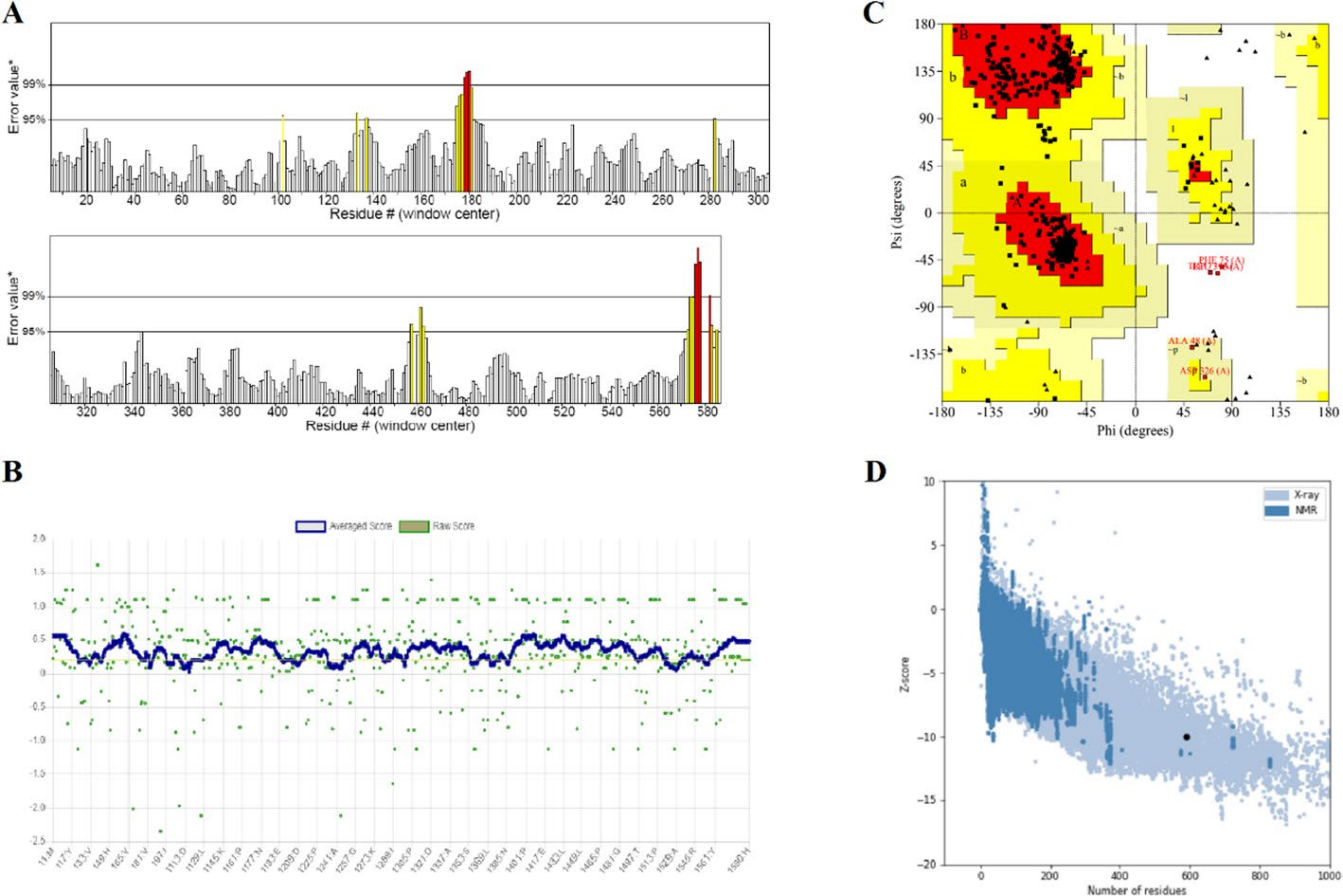
Validation of the refined model of the multi-epitope vaccine. **A** The ERRAT quality factor of the refined model was estimated as 96.00. **B** The VERIFY-3D value of the refined model was calculated at 83.39. **C** Ramachandran plot of the refined model of vaccine showed 94.2%, 5.1% and 0.6% of residues in the favored, allowed, and disallowed regions, respectively. (D) Z-score of the 3D-structure of the refined model of vaccine was estimated as − 10.02, which lay in the range of scores reported for native protein structures

The tertiary structure of the designed multi-epitope construct was predicted by the Robetta server using a comparative modeling approach. The generated model number one was refined by the GalaxyRefine server. Among the five refined models, model number 1 (Fig. 2C) was selected as the best structure for further analysis. This model had a GDT-HA score of 0.9712, RMSD score of 0.380, MolProbity score of 1.591, clash score of 8.4, Poor rotamers of 0.2 and Rama favored score of 97.3. The crude and refined models were evaluated using the programs such as ERRAT, VERIFY-3D and PROCHECK from SAVES server, and also ProSA web server (Table 3). The ERRAT quality factor score of the crude model was predicted as 96.30, while the score of the refined model was calculated as 96.00 (Fig. 3A). VERIFY-3D value of the primary model was estimated as 80.68, which was increased to 83.39 in the refined model (Fig. 3B). The Ramachandran Plot produced by PROCHECK demonstrated that in the crude model, 91.4%, 8.3% and 0.2% of residues were in the favored, allowed and disallowed regions, respectively, while in the refined model, 94.2%, 5.1% and 0.6% of residues were in the favored, allowed and disallowed regions, respectively (Fig. 3C). The Z-score obtained by the ProSA was found to be − 9.83 in the crude model compared to − 10.02 in the refined model (Fig. 3D). The results indicate that the final model has definitely a good quality.

### IFN-inducing peptide prediction in the final proposed construct

The IFNepitope server was used to identify the epitopes that can activate T-helper cells for Interferon gamma production. A total number of 105 and 464 potential IFN-γ epitopes were predicted for the Ply4 adjuvant candidate and the main vaccine sequence, respectively. The positive epitopes with scores greater than or equal to one, a total of 29 and 45 epitopes from the Ply4 and the main vaccine sequence, respectively, are presented in Table 4.

### Prediction of conformational B cell epitopes of the final designed protein

**Fig. 4:**
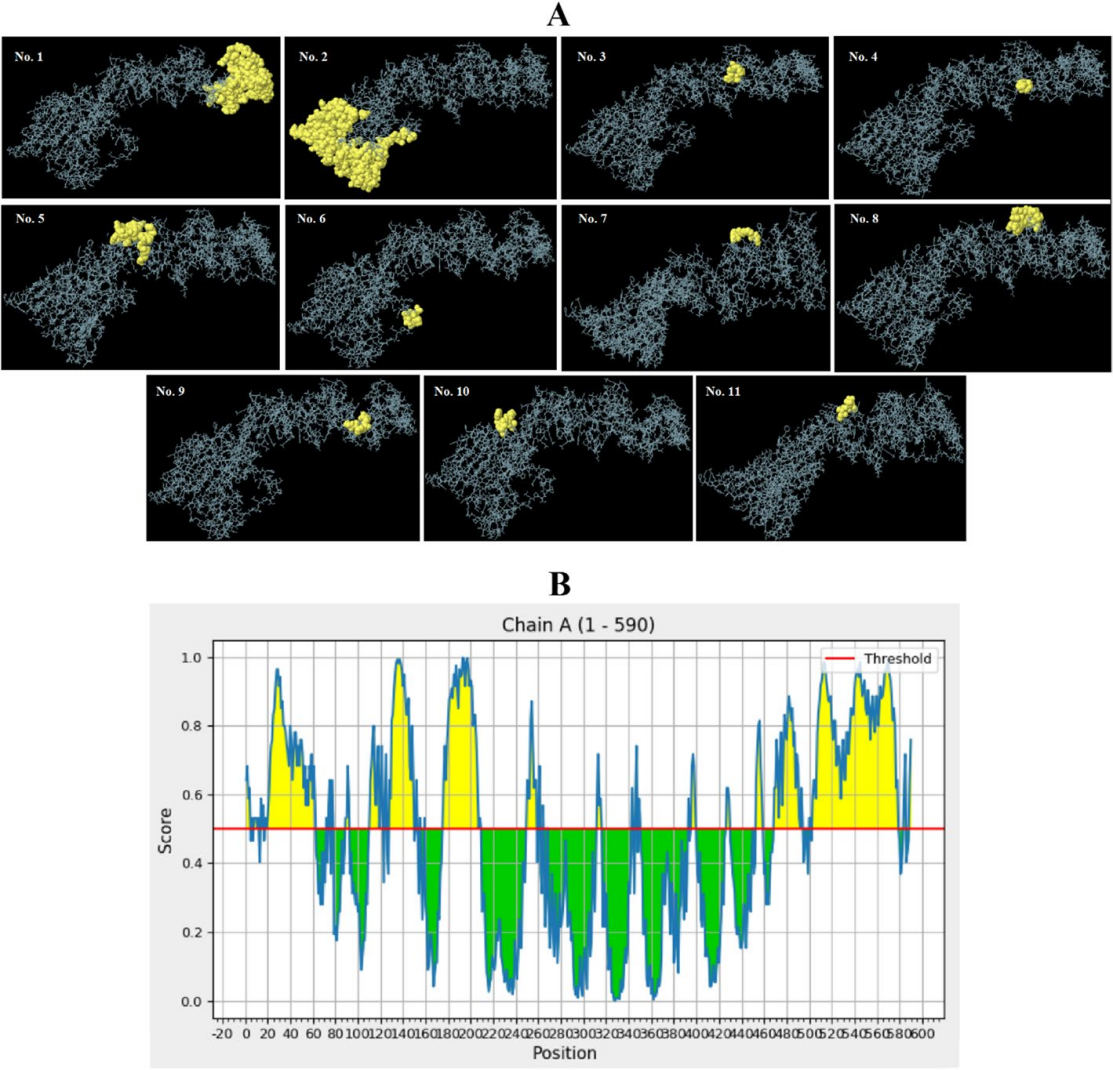
3D images of the conformational B cell epitopes of the designed vaccine and 2D score chart. **A** The yellow and grey regions represent the conformational epitopes and the bulk of the polyprotein, respectively. **B** The yellow parts with a score above the threshold 0.5 display potential B cell epitopes

Since greater than 90% of B-cell epitopes are conformational/discontinuous, the 3D structure of the proposed multi-epitope construct was analyzed to identify these epitopes via the ElliPro server. Eleven new epitopes, comprising 3–114 residues were found with a score value of 0.518–0.776. The details of the identified conformational epitopes are given in Table 5. The 3D representation of the epitopes in the final construct and the 2D score chart are shown in Fig. 4 A and B, respectively.

### Vaccine-TLR-4 docking

**Fig. 5:**
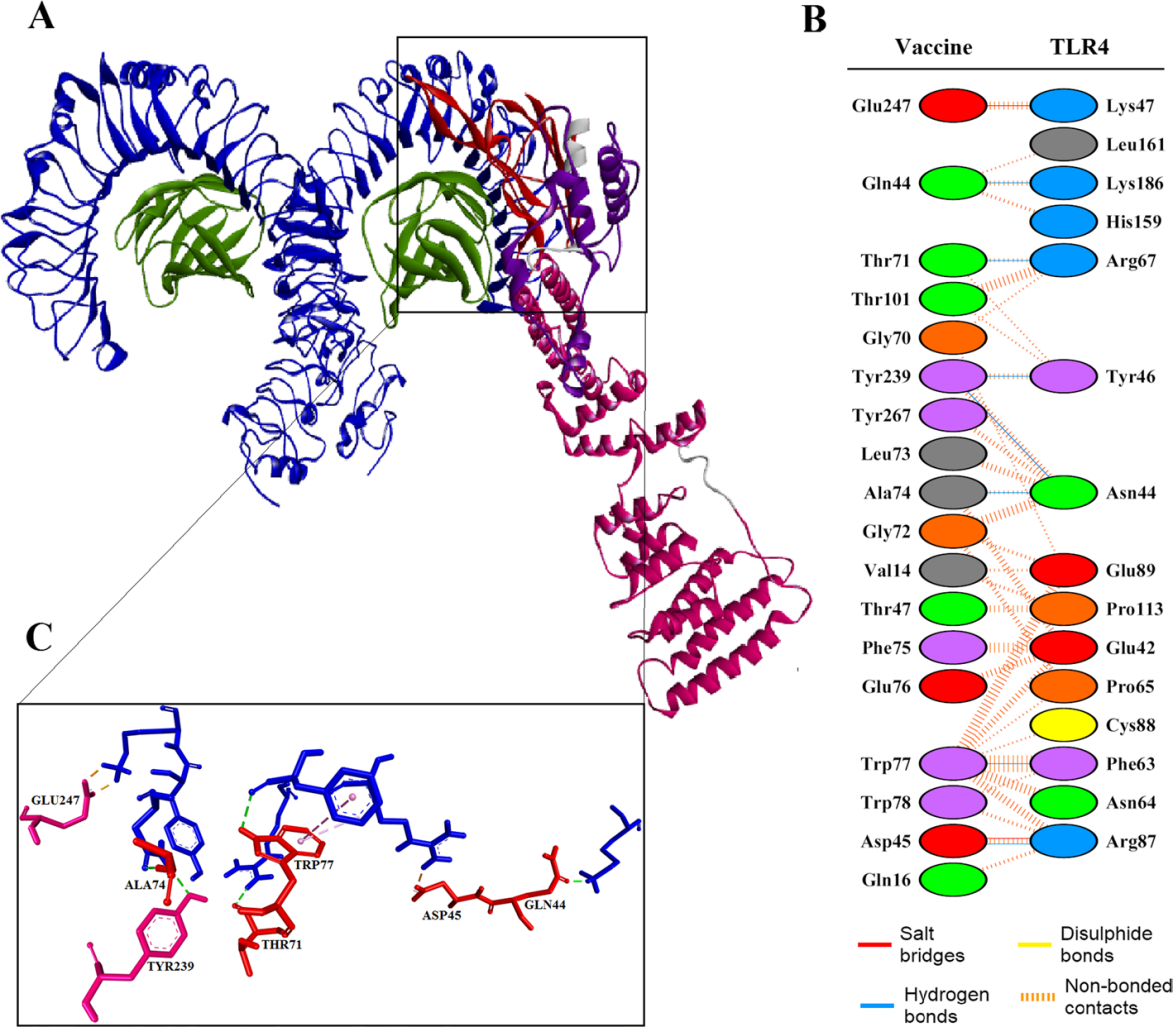
Molecular docking of the designed vaccine with the TLR4 receptor. **A** The cartoon representation of the vaccine-TLR4 complex visualized by Discovery Studio Visualizer. **B** A number of 18 residues of the whole vaccine candidate paired with 15 residues of the TLR-4 ECD. The whole vaccine established 2 salt bridges, 7 hydrogen bonds and 116 non-bonded contacts with the receptor. **C** The residues involved in interactions among the vaccine and TLR4 ECD are represented as a stick model and colored in red/magenta and blue, respectively, while the vaccine residues are labeled and the interactions are shown with dashed lines

Molecular docking of the refined model of vaccine with the TLR4 protein was done, and a total of 30 models were ranked based on the amount of energy produced using the ClusPro 2.0 server. The model M02 as the best-docked complex displayed the center weighted score of − 740.8 kcal/mol and lowest energy score of − 809.3. As shown in Fig. 5, the TLR4 receptor and the vaccine protein interacted significantly through the domain 4 of Ply as the TLR ligand. PDBsum revealed that 15 residues of Ply4 were paired with 14 residues of the extracellular domain (ECD) of TLR4. The Ply4 established one salt bridge, five hydrogen bonds and 100 non-bonded contacts with the ECD of TLR4 (Fig. 5B). The salt-bridge between Ply4 and TLR4 ECD was formed among the residues Asp45 and Arg87, respectively, as well as the hydrogen bonds were formed between Ala74-Asn44, Trp77-Phe63, Thr71-Arg67, Asp45-Arg87 and Gln44-Lys186, at a distance of 3.01, 2.71, 2.93, 2.76 and 2.59 Å, respectively. This complex was applied as the initial configuration for the process of molecular dynamics simulation.

### Molecular dynamics (MD) simulation of the vaccine in complex with the immune receptor

**Fig. 6:**
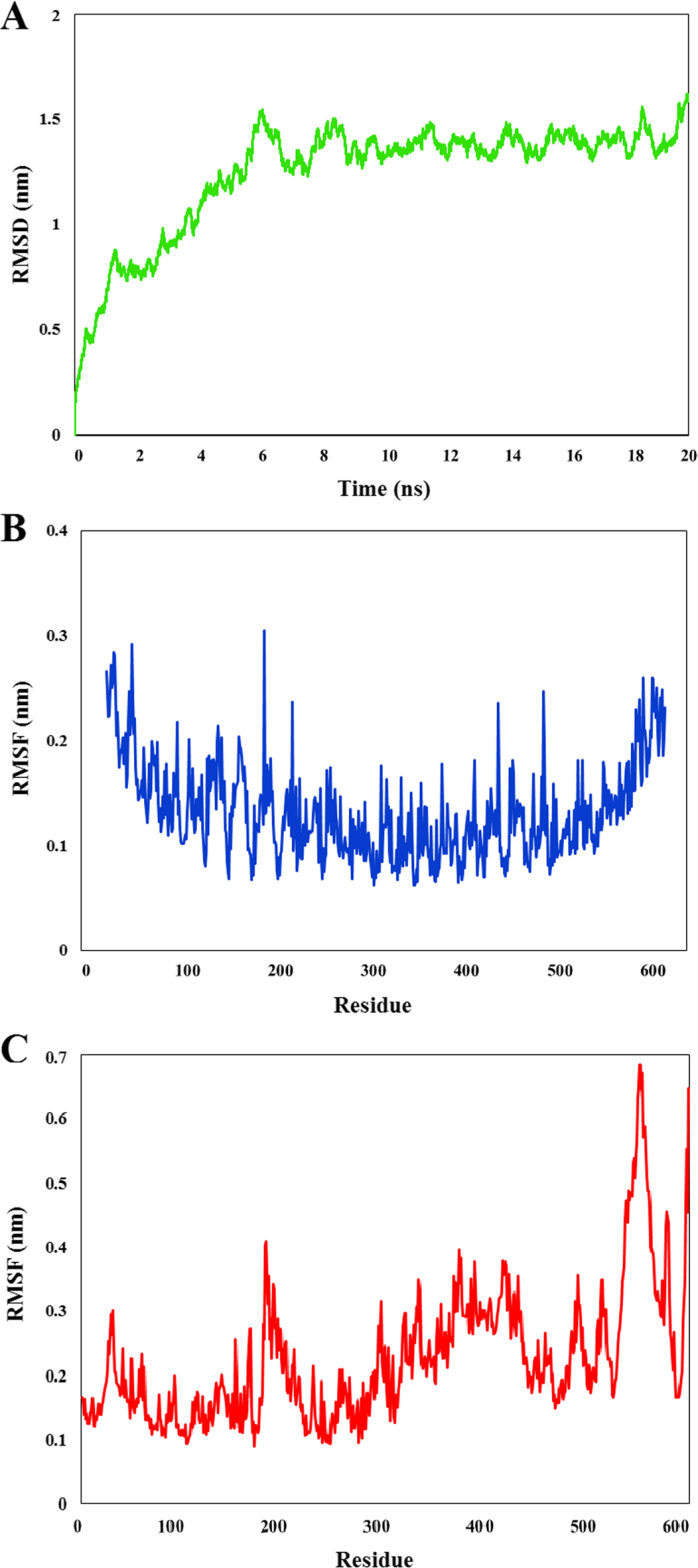
The results of MD simulation of the vaccine-TLR4 docked complex. **A** RMSD values for the vaccine-receptor complex during a period of 20 ns. The complex became stable after 6 ns of MD simulation, which further confirmed the interaction between the vaccine and TLR4. **B** and **C** show the RMSF of all the residues of construct and TLR4 ECD, respectively. Most of the receptor residues underwent small fluctuations. Two regions of the vaccine indicated relatively high fluctuations, showing relatively high flexibility

The stability of the designed vaccine in complex with TLR4 ECD was analyzed by performing MD simulation, and the results were evaluated in terms of RMSD and RMSF. The RMSD profile displayed the changes in backbone Cα atoms from the initial to final conformations of the protein complex. RMSD values of the vaccine-receptor complex indicated a sharp increase from the starting point of the simulation up to 6 ns, and after that until 20 ns remained steady with small fluctuations at an average of slightly lower than 1.5 nm (Fig. 6A). The calculations of RMSF of Cα atoms determined the flexibility of all residues in the vaccine protein and TLR4 ECD. The results indicated that the most amino acids in the TLR4 ECD had small fluctuations and the minimum and maximum RMSF values were 0.1 and 0.3, respectively (Fig. 6B). Two regions of the vaccine molecule including amino acids in areas 179–181 and 551–555 showed relatively high fluctuations with RMSF of ~ 0.4 and 0.68 nm, respectively, indicating relatively high flexibility (Fig. 6C).

### Reverse translation, codon optimization, and in silico cloning

**Fig. 7:**
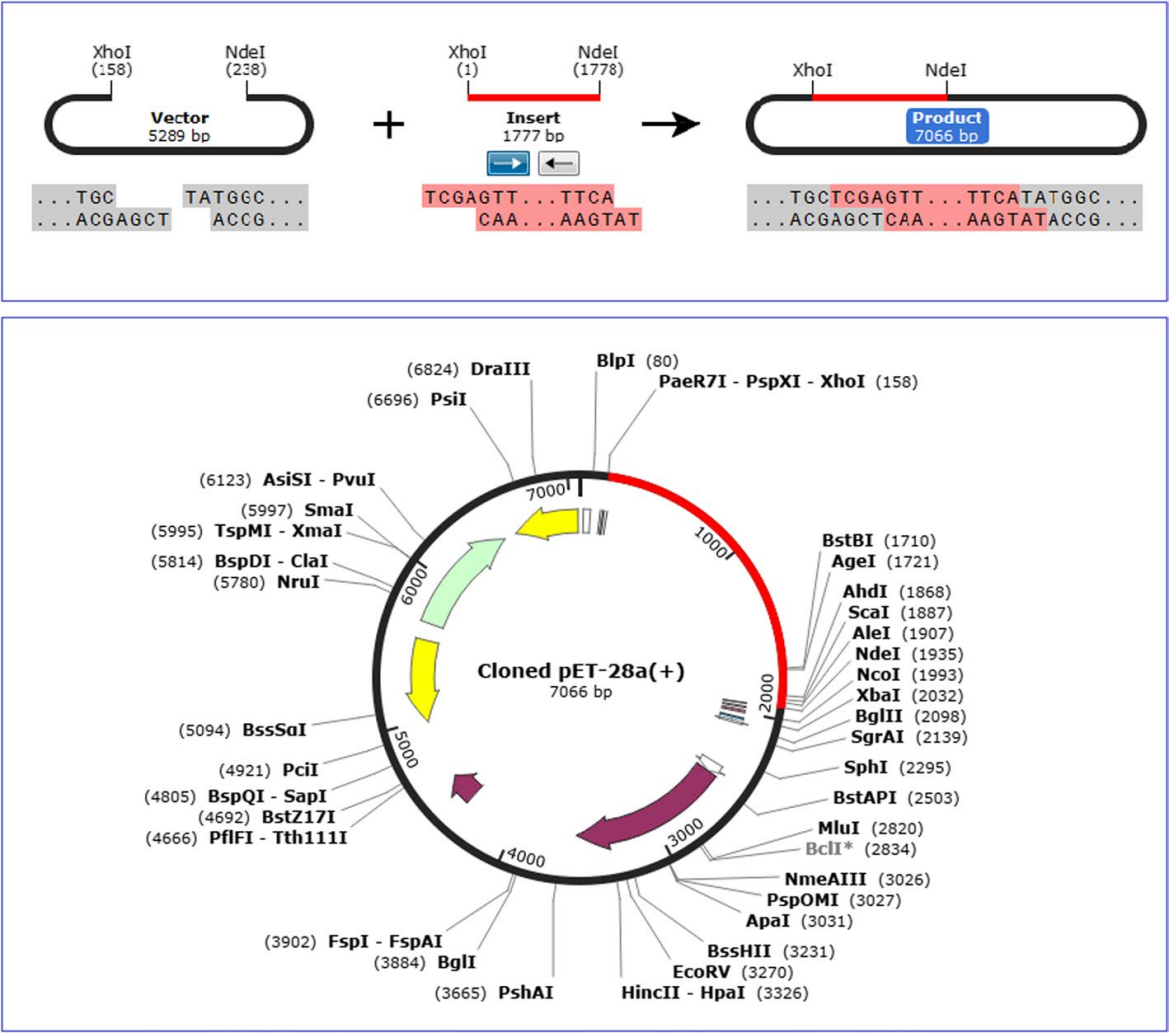
In silico cloning of the target sequence in pET28a(+) vector plasmid. The vaccine sequence between the restriction sites is represented in red color while the vector backbone is shown in black. The total length of the clone is 7066 bp

The codon adaptation of the designed vaccine construct was done using JCat server with respect to the codon usage of *E. coli* strain K12. The total length of the target sequence was 1770 nucleotides. The Codon Adaptation Index (CAI) of the improved sequence was found to be 0.99, which is near to 1.0 indicating high level expression of the construct in this bacterial system. The GC-content was predicted to be 53.27%, which is within the optimal range (30–70) and shows a high probability of expression in the *E. coli* K12 strain (Additional file 1: Fig. S6). The adapted vaccine sequence was then inserted into the pET28a(+) expression vector between the restriction enzymes *Nde*I and *Xho*I using the SnapGene tool (Fig. 7).

## Discussion

Pneumococcal infections are responsible for substantial morbidity and mortality, particularly in children, and it is believed that an effective vaccine can reduce the death rate [50, 51]. Since the existing polysaccharide-based vaccines are costly, serotype-dependent, and their immunogenicity is restricted to the serotypes included in the vaccines, the efforts of scientists have been focused on the development of serotype-independent vaccines [52]. Pneumococcal protein vaccines are currently being evaluated as interesting alternatives to available vaccines, that can induce serotype-independent immunity at a low cost [16–18]. The combination of multiple protein antigens in a vaccine seems like a very attractive strategy to block infection by targeting several important virulence factors [53]. Various studies have shown that a fusion protein comprising multiple antigens can be more effective than a mixed formulation, and also relatively simplify the purification procedure and facilitate product quality control [54–56].

Recent advances in immunoinformatics approaches could help to identify potential B- and T-cell epitopes on antigenic proteins for the construction epitope-based subunit vaccines and so to accelerate the vaccine development process [57–59]. In recent years, numerous researchers have designed novel vaccines using immunoinformatics tools against different pathogens like *Acinetobacter baumannii* [60], *Listeria monocytogenes* [61], *Leishmania donovani* [62], influenza A virus [63], and HPV16/18 [64]. Epitope-based peptide vaccines have many advantages compared to vaccines made by conventional technologies. For instance, they are a safer alternative, as they do not contain the complete pathogen and are more stable and highly specific. They have lower costs, do not require microbial culturing, and reduce the number of in vitro experiments, saving time [65]. Nevertheless, epitope vaccines have some limitations, in particular low immunogenicity due to their quick degradation by peptidases and lack of detection by immune receptors [66]. To conquer this issue several approaches can be employed such as developing adjuvanted vaccines that can improve the targeting of antigens to antigen-presenting cells and so prevent degradation of the antigen in vivo [67, 68].

Bacterial protein TLR agonists have been studied as attractive vaccine adjuvant candidates in recent decades, including several pneumococcal proteins, such as pneumolysin [40, 41]. A study from Chiu et al. has demonstrated that the truncated domain 4 of pneumolysin alone has two activities, stimulating TLR4 and causing hemolysis [43]. Besides, It has been shown that a triple mutant comprising Asp-385 to Asn, Cys-428 to Gly and Trp-433 to Phe substitutions could reduce cytolytic activity of the Ply protein [69]. Therefore, the domain 4 of Ply, along with the mutations D385N, C428G and W433F, could be considered as a potential candidate to be used as a TLR agonist adjuvant in order to interact with TLR4 and substantially increase immune responses to genetically fused peptide antigens. In this study, it was attempted to employ computational approaches for engineering a potential Ply4-adjuvanted multi-epitope vaccine comprising the immunodominant regions of PspAs from different clades and PhtD, as the most promising antigens, to trigger neutralizing antibodies against different strains of *S. pneumoniae*.

Numerous studies have demonstrated that recombinant PspAs could protect animal models from fatal pneumococcal infection [26, 70, 71] and also elicit strong antibody responses in human phase I clinical trials [72, 73]. However, the diversity of PspAs among clinical isolates could limit the coverage of PspA-based vaccines [30]. Based on analysis of amino acid sequence divergence, Hollingshead et al. classified most PspAs into two major families divided into 5 clades [72]. It has been shown that the cross-reactivity level between PspAs is dependent on the sequence identity, which is low among different PspA families and higher within each family. Moreover, some studies have suggested that the cross-reactivity/protection level differ depending on the PspA clade [30–32]. Akbari et al. developed a fusion PspA-based vaccine containing B region fragments of PspA from the five most prevalent clades (named: PspAB1-5). The vaccine could provide significant protection against different strains of pneumococci, indicating that the use of fusion proteins bearing B regions from clades 1–5 could be a promising immunization strategy. Moreover, since the A and C regions also play a significant role in the production of cross-reactive antibodies, it was suggested that including these regions in the construct could be helpful to expand the cross-protection of the vaccine [32]. In this respect, in the current study, the regions A, B, and C of PspA were evaluated for prediction of their B- and helper T-cell epitopes. B regions from clades 1–5 were considered in order to provide broad coverage and reduce the likelihood of PspA variants escaping host immunity.

The PhtD protein has been shown to induce a broad protection against colonization and lethal infections in animal models [17, 74], and also to be safe and immunogenic in phase I clinical trials, rendering promising outcomes [18]. The studies have indicated that the N-terminal amino acids of PhtD are involved in surface attachment, while the C-terminal fragment of PhtD is a surface-exposed and protection-eliciting region [37, 75]. In a study by Plumptre et al., recombinant truncated derivatives of the C-terminal of PhtD were found to induce a very high titre of antibodies, showing that the region was highly immunogenic, while the full length PhtD was ineffective in providing significant protective immunity [37]. Therefore, herein, we analyzed the C-terminal of PhtD as a favorable immunogenic fragment to predict the B-cell and helper T-cell epitopes.

Based on the immunoinformatics results obtained in this research, the high-scoring B- and T-cell epitopes overlapping with each other shared between different servers were considered, and the final peptides from PspA (A/B region) and PhtD-C antigens were selected (Table 1). A comparative evaluation of the computationally predicted epitopes with the existing experimentally validated epitopes of PspA was done to control the accuracy of the present in silico prediction. The considered peptides of B region of PspA1 overlapped with the sequences ESDSEDYVK and SDGEQAGQYLAAAEE reported as B-cell epitopes by Vadesilho et al. [24]. Also, one of the selected peptides of B region of clade3 was consistent with the experimentally identified epitope sequence DSEDDTAA. The chosen peptides for B regions of clades 4 and 5 overlapped with the epitope sequences NNVEDYIKEG and NNVEDYVKEG reported as B-cell epitopes by the same research group [24]. Further to these, the considered peptide with amino acids 190–215 of B region of clade 2 overlapped with the sequence EDYAKEGFRAPLQSK experimentally obtained as T-cell epitope by Singh et al. [76]. These further demonstrated the accuracy of the current computational prediction. For epitope prediction of C region of PspA, experimentally identified epitopes available on the IEDB server were used. The sequences of C region in different PspAs are mainly composed of short amino acid repeats [27]. It has been found that 46% of strains express a copy of the short repeat PKPEQP which is able to elicit protective antibodies in mice [23], therefore this significant motif was considered in this research. In the next step of this study, the screened peptides were linked to each other using appropriate linker, namely GPGPG, as flexible glycine-rich linkers can improve the solubility and allow the adjoining domains to be accessible and function freely [77, 78]. To generate whole vaccine, the sequence of Ply4, the considered potential TLR-4 agonist candidate, was added to the N-terminal end of the fused peptides using the EAAAK, as this linker provides rigidity and reduce possible interference of other regions of the protein in the interaction between the adjuvant and its receptor [79].

As shown in Table 2, physicochemical and immunological features of the construct were appraised using various computational servers. The molecular weight of the vaccine was 62.96 kDa, which makes it an acceptable construct because proteins with a molecular weight of less than 110 kDa can be more easily purified and so are thought to be more suitable targets for the vaccine development [80]. Also, this indicator is useful in SDS-PAGE electrophoresis and Western blot assays [81]. The theoretical pI of the vaccine was found to be rather acidic in nature (4.60), which could be beneficial for isoelectric focusing and purification of protein by ion exchange chromatography [81]. The half-life of the vaccine was found to be > 10, > 20, and 30 h in *E. coli*, yeast and mammalian, respectively, which shows the time taken by the protein to reach half of its amount after being synthesized in the cell [82]. The recombinant vaccine with instability index of 33.71 was classified as stable, since proteins with instability index less than 40 are considered to be stable and vice versa [82]. The calculated aliphatic index of multi-epitope protein was 59.63, indicating that the construct was thermostable [83]. The GRAVY index of the vaccine was a negative value (− 1.028), which indicated hydrophilic nature of the protein and its strong interaction with water molecules, suggesting high solubility [84]. For an antigen, greater interactions with water molecules reflect greater interactions with elements of the immune system, particularly with B cell receptors/antibodies [85]. Additionally, the analysis of solubility showed that the protein has a high percentage of solubility upon overexpression in *E. coli*. The antigenicity of the designed vaccine was evaluated to be 0.8833 and 0.850188 using VaxiJen and ANTIGENpro servers, respectively, showing the protein is antigenic in nature. The vaccine candidate was predicted to be a non-allergen molecule by two web servers AlgPred and AllerTOP.

**Table 2:** Physicochemical and immunological properties of the candidate vaccine

Molecular features	Prediction
No. of amino acids	590
Molecular weight (kDa)	62.96
Theoretical pI	4.60
Total no. of negatively charged residues (Asp + Glu)	115
Total no. of positively charged residues (Arg + Lys)	64
Estimated half-life	> 10 h (*Escherichia coli*, in vivo)
	> 20 h (yeast, in vivo)
	30 h (mammalian reticulocytes, in vitro)
Instability index	33.71 (Stable)
Aliphatic index	59.63
Grand average of hydropathicity (GRAVY)	− 1.028
Solubility (SOLpro)	0.958393 (Soluble)
Antigenicity (VaxiJen)	0.8833 (Probable ANTIGEN)
Antigenicity (ANTIGENpro)	0.850188 (Probable ANTIGEN)
Allergenicity (AlgPred/AllerTOP)	Non-allergen

The initial 3D structure of the vaccine candidate modeled by Robetta server was subjected for refinement, by GalaxyRefine software, to obtain a more high-quality 3D model. The evaluation of the initial and refined structures was performed using Ramachandran plot, ProSA Z-score, Errat score and Verify3D score. The obtained results showed that the quality of tertiary structure of vaccine has been improved by refinement (Table 3). The Ramachandran plot after refinement indicated that most of the residues were found in the favoured regions (94.2%), showing that the quality of the model was satisfactory. In addition, after refinement, the Z-score of − 10.02, ERRAT score of 96.00 and Verify3D score of 83.39 further validated the overall quality of the multi-epitope vaccine.

**Table 3:** Evaluation results of the modeled vaccine before and after refinement. Comparisons were performed by various servers ERRAT, Verify3D, PROCHECK, and ProSA

Server	Before refinement	After refinement
ERRAT	96.30	96.00
Verify3D	80.68	83.39
PROCHECK	Most favoured regions, 91.4%	Most favoured regions, 94.2%
	Additional & generously allowed regions, 8.3%	Additional & generously allowed regions, 5.1%
	Disallowed regions, 0.2%	Disallowed regions, 0.6%
ProSA	− 9.83	− 10.02

Identification of interferon-γ inducing peptides and also conformational B-cell epitopes was performed after the evaluation and confirmation of the construct. IFNepitope server predicted 74 epitopes (Table 4) in the construct's sequence that could induce IFN-γ which has been reported as one of essential factors involved in combating fatal pneumococcal infection [86]. The ElliPro server predicted eleven potential non-linear B-cell epitopes in the final 3D model of vaccine with 3–114 residues and qualifying scores of 0.518–0.776 (Table 5). The results confirm that the vaccine is capable to stimulate the humoral immune response which is essential for protection against pneumococcus. Since the TLR4 agonist Ply4 was used as a potential immune-adjuvant in the vaccine, a molecular docking analysis was performed to assess potential immune interaction between the multi-epitope vaccine and TLR4 (Fig. 5). The results confirmed that the designed construct via Ply domain 4 had affinity for TLR4, with the lowest energy scores of − 809.3. The docking analysis indicated that 2 salt bridges and 7 hydrogen bonds were formed during the interaction. To check the stability of vaccine-TLR4 complex, a molecular dynamics simulation was done using the best docked model. According to Fig. 6A, there were very mild fluctuations in the RMSD graph from 6 until 20 ns, suggesting the complex’s stability. The RMSF graphs showed the most residues underwent small fluctuations, except two regions of the vaccine (Fig. 6B–C). Based on the obtained results, our developed vaccine can maintain a sustained interaction with TLR4 and thus has the chance to induce potentially protective immune responses. To assure a high-level expression in *E. coli* (strain K12), codon optimization of the proposed construct was performed using Jcat server. The expression of protein correlates with the total GC content and CAI value of the optimized reverse translated sequence [87]. Both results of the GC content (53.27%) and CAI value (0.99) were favorable for high-level expression of the multi-epitope protein in the bacteria. Finally, the vaccine sequence was successfully ligated into the pET28a( +) expression vector for in silico cloning. The results of immunoinformatics analysis of the vaccine candidate demonstrated that this construct may have a high potency against pneumococcus, but further wet laboratory studies are needed in vitro and in vivo to confirm these results.

**Table 4:** The predicted IFN-γ epitopes from the proposed construct. The hybrid approach (MERCI and SVM) were used to predict IFN-gamma epitopes by the IFNepitope server. The results revealed that there were 29 and 45 positive epitopes with scores greater or equal to 1 for the Ply4 adjuvant candidate and the main sequence of vaccine, respectively. The sequences were sorted from higher to lower scores

No.	Sequence	Method	Score	No.	Sequence	Method	Score
*The candidate adjuvant*							
1	AYVAQYYITWDELSY	MERCI	5	16	GTGLAFEWWRTVYEK	MERCI	2
2	YVAQYYITWDELSYN	MERCI	5	17	TGLAFEWWRTVYEKT	MERCI	2
3	VAQYYITWDELSYNH	MERCI	5	18	NGQDLTAHFTTSIPL	MERCI	1
4	AQYYITWDELSYNHQ	MERCI	5	19	GQDLTAHFTTSIPLK	MERCI	1
5	QYYITWDELSYNHQG	MERCI	5	20	QDLTAHFTTSIPLKG	MERCI	1
6	YYITWDELSYNHQGK	MERCI	5	21	DLTAHFTTSIPLKGN	MERCI	1
7	YITWDELSYNHQGKE	MERCI	5	22	LTAHFTTSIPLKGNV	MERCI	1
8	IREGTGLAFEWWRTV	MERCI	3	23	TAHFTTSIPLKGNVR	MERCI	1
9	REGTGLAFEWWRTVY	MERCI	3	24	AHFTTSIPLKGNVRN	MERCI	1
10	GAYVAQYYITWDELS	MERCI	2	25	RNLSVKIREGTGLAF	MERCI	1
11	LSVKIREGTGLAFEW	MERCI	2	26	NLSVKIREGTGLAFE	MERCI	1
12	SVKIREGTGLAFEWW	MERCI	2	27	GLAFEWWRTVYEKTD	MERCI	1
13	VKIREGTGLAFEWWR	MERCI	2	28	LAFEWWRTVYEKTDL	MERCI	1
14	KIREGTGLAFEWWRT	MERCI	2	29	AFEWWRTVYEKTDLP	MERCI	1
15	EGTGLAFEWWRTVYE	MERCI	2				
*The main vaccine*							
1	TPPSTDHQDSGNTEA	MERCI	3	24	ESEDYAKEGFRAPLQ	MERCI	2
2	PPSTDHQDSGNTEAK	MERCI	3	25	SEDYAKEGFRAPLQS	MERCI	2
3	PSTDHQDSGNTEAKG	MERCI	3	26	EDYAKEGFRAPLQSK	MERCI	2
4	STDHQDSGNTEAKGA	MERCI	3	27	EQPAGPGPG	SVM	1.08
5	TDHQDSGNTEAKGAE	MERCI	3	28	PGPGVQQAYLAYQRA	SVM	1.03
6	DHQDSGNTEAKGAEA	MERCI	3	29	GLTPPSTDHQDSGNT	MERCI	1
7	LTPPSTDHQDSGNTE	MERCI	2	30	QDSGNTEAKGAEAIY	MERCI	1
8	HQDSGNTEAKGAEAI	MERCI	2	31	LDRMPYNLQYTVEVK	MERCI	1
9	KKSEAAKKAYEGPGP	MERCI	2	32	DRMPYNLQYTVEVKN	MERCI	1
10	KSEAAKKAYEGPGPG	MERCI	2	33	RMPYNLQYTVEVKNG	MERCI	1
11	SEAAKKAYEGPGPGV	MERCI	2	34	MPYNLQYTVEVKNGS	MERCI	1
12	EAAKKAYEGPGPGVQ	MERCI	2	35	NPSAGPGPGSPVASQ	MERCI	1
13	AAKKAYEGPGPGVQQ	MERCI	2	36	EDYVKEGFRAPLQSE	MERCI	1
14	AKKAYEGPGPGVQQA	MERCI	2	37	DYVKEGFRAPLQSEL	MERCI	1
15	PGLKEIDESESEDYA	MERCI	2	38	YVKEGFRAPLQSELD	MERCI	1
16	GLKEIDESESEDYAK	MERCI	2	39	VKEGFRAPLQSELDG	MERCI	1
17	LKEIDESESEDYAKE	MERCI	2	40	KEGFRAPLQSELDGP	MERCI	1
18	KEIDESESEDYAKEG	MERCI	2	41	EGFRAPLQSELDGPG	MERCI	1
19	EIDESESEDYAKEGF	MERCI	2	42	DPEDDTAALQNKGPG	MERCI	1
20	IDESESEDYAKEGFR	MERCI	2	43	PEDDTAALQNKGPGP	MERCI	1
21	DESESEDYAKEGFRA	MERCI	2	44	EDDTAALQNKGPGPG	MERCI	1
22	ESESEDYAKEGFRAP	MERCI	2	45	DDTAALQNKGPGPGK	MERCI	1
23	SESEDYAKEGFRAPL	MERCI	2

**Table 5:** Conformational/discontinuous B-cell epitopes in the final construct predicted by Ellipro server

No.	Residues	No. of residues	Score
1	G453, P454, G455, P456, G457, K458, V459, L460, A461, G467, K468, T469, Q470, D471, E472, L473, D474, K475, E476, A477, A478, E479, A480, G481, P482, G483, P484, G485, S486, K487, L488, E489, D490, N491, L492, K493, D494, T497, V500, E501, Y503, I504, K505, E506, G507, G508, P509, G510, P511, G512, P513, E514, G515, K516, T517, Q518, D519, E520, L521, D522, K523, E524, A525, A526, E527, D528, A529, N530, I531, E532, A533, L534, Q535, N536, K537, V538, A539, G540, P541, G542, P543, G544, R545, L546, Q547, S548, D549, L550, K551, D552, A553, E554, E555, N556, N557, V558, E559, D560, Y561, V562, K563, E564, G565, L566, G567, P568, G569, P570, G571, A572, P573, K574, P575, E576	114	0.776
2	M1, N2, G3, D4, L6, D8, H9, S10, G11, A12, Y13, V14, A15, Q16, Y18, I19, T20, W21, D22, E23, L24, S25, Y26, N27, H28, Q29, G30, K31, E32, V33, L34, T35, P36, K37, A38, W39, D40, R41, N42, G43, Q44, D45, L46, T47, A48, H49, F50, T51, T52, S53, I54, P55, L56, K57, G58, N59, V60, R61, N62, P88, L89, V90, R91, K92, T100, V110, E111, N112, D113, E114, A115, A116, A117, K118, A119, A120, Q121, A122, A124, K125, G128, L129, T130, P131, P132, S133, T134, D135, H136, Q137, D138, S139, G140, N141, T142, E143, A144, K145, G146, A147, E148, A149, Y151, N152, K155, V173, E174, V175, K176, N177, G178, S179, G180, P181, G182, P183, G184, K185, P186, Q187, T188, E189, K190, P191, E192, E193, D194, K195, E196, H197, D198, E199, V200, S201, E202, P203, T204	137	0.734
3	H586, H589, H590	3	0.602
4	P583, G584, H585	3	0.601
5	A252, Y253, E254, G255, P256, G257, P258, V260, Q261, Y264, G312, P313, G314, P315, G316, K317, G340, G342, P343, G344, K346	21	0.589
6	H205, P206, E207, S208, D209	5	0.587
7	G426, P427, G428, P429, G430	5	0.559
8	Y390, T392, E393, G394, L395, E396, K397, G398, P399, G400, P401	11	0.534
9	E466, Q577, P578, A579, G580	5	0.532
10	T71, G72, L73, A74, F75, W77	6	0.522
11	I348, D349, E350	3	0.518

## Conclusion

The purpose of this research was to design a protective, antibody-inducing, multi-epitope vaccine against *S. pneumoniae*. To achieve this aim, the immunodominant B- and helper T-cell epitopes from highly conserved and protective antigens of pneumococcus, i.e., PspA (clades 1–5) and PhtD, were predicted computationally and the final considered regions were linked together using suitable linkers. The domain 4 of Ply as a potential adjuvant candidate was incorporated into the multi-epitope construct to improve the immunogenicity of epitope vaccine and to induce and increase the humoral immune responses against pneumococcus. After evaluation of the physicochemical properties, antigenicity and allergenicity of the final construct, its 3D structure was predicted by online tool. Structural quality, binding affinity to TLR4, and stability of the vaccine-receptor complex were analyzed. Finally, codon optimization was performed for gene expression in *E. coli* and in silico cloning was performed in the pET28a(+) vector. In spite of the significant results of this study obtained using in silico analysis, further experimental evaluation of the suggested multi-epitope peptide vaccine candidate is needed to confirm its efficacy.

## Materials and methods

### Amino acid sequence retrieval

The NCBI protein database (www.ncbi.nlm.nih.gov/protein) was used for the retrieval of the protein sequences of PspA from strains DBL6A (Clade 1) [AC AAF27701], WU2 (Clade 2) [AC AAF27710], BG8090 (Clade 3) [AC AAF27713], EF5668 (Clade 4) [AC AAC62252] and ATCC6303 (Clade 5) [AC AAF27715], as well as PhtD from strain R6 [AC AAK99711] and Ply from strain D39 [AC ABJ53672]. The proteins PspA and PhtD were used for prediction of B-cell and helper T-cell epitopes and pneumolysin was applied as a TLR4 agonist.

### Prediction of transmembrane regions

The presence of the transmembrane regions of the proteins PspA and PhtD were evaluated using TMHMM 2.0 (www.cbs.dtu.dk/services/TMHMM-2.0) [88]. TMHMM, which is based on a hidden Markov model, can discriminate between membrane and soluble proteins with a high degree of accuracy.

### Modeling and model validation of candidate proteins

In order to predict the conformational epitopes, the protein tertiary structures were constructed by I-TASSER server (https://zhanglab.ccmb.med.umich.edu/I-TASSER/) [89] or SWISS-MODEL server (http://swissmodel.expasy.org) [90] with respect to the presence of homologous templates with sequence identity less or more than 30%, respectively. The refinement of low-quality structures was done using the servers ModRefiner (https://zhanglab.ccmb.med.umich.edu/ModRefiner/) [91] and GalaxyRefine (http://galaxy.seoklab.org/cgi-bin/submit.cgi?type=REFINE) [92]. The 3D structure validation was conducted using the programs PROCHECK [93] and ERRAT [94] at SAVES server version 6 (https://saves.mbi.ucla.edu/). PROCHECK checks the stereochemical quality of 3D models by plotting the Ramachandran plot [93], and ERRAT validates the structures through statistical analysis of non-bonded interactions among different types of atoms [94]. BIOVIA Discovery Studio Visualizer was utilized for viewing the generated molecular structures.

### Identification of linear/conformational B cell epitopes

Linear B-cell epitopes were identified by the servers LBtope (http://crdd.osdd.net/raghava/lbtope/) [95], ABCpred (http://crdd.osdd.net/raghava/abcpred/) [96], Emini surface accessibility prediction tool of IEDB (http://tools.iedb.org/bcell/result/) [97] and Ellipro at IEDB sever (http://tools.iedb.org/ellipro/) [98]. LBtope server, which is based on SVM, has a predictive accuracy of ~ 81%. This server assigns scores between 0 and 100 percent to each of the predicted epitopes and higher score signifies a higher probability of being an epitope. ABCpred, based on neural networks, predicts linear epitopes with an overall accuracy of 65.93%. A score closer to one corresponds a higher likelihood of the sequence being an epitope. Emini method was utilized to predict surface-accessible epitopes holding the default threshold value 1.0.

Furthermore, conformational epitopes from the modeled structures were predicted by the softwares ElliPro and DiscoTope 2.0 (http://tools.iedb.org/discotope/) [99]. Ellipro server identifies linear and structural epitopes based on solvent-accessibility and flexibility. This server predicts the antibody epitopes based on a three-step process: (1) approximation of the surface of the protein as an ellipsoid, (2) computation of the protrusion index (PI) for each protein residue and (3) clustering of neighboring protein residues on the basis of the PI values. The higher scores correspond to the larger solvent accessibility. DiscoTope 2.0 sever predicts discontinuous epitopes in 3D protein structures based on half-sphere exposure and propensity scores, with the default threshold − 3.7 (corresponding to sensitivity 0.47 and specificity 0.75).

### Helper T cell (MHC-II) epitope prediction

Identification of binding peptides to MHC-II molecules was performed by the consensus method of IEDB MHC-II prediction tool (http://tools.immuneepitope.org/mhcii) [100] and NetMHCIIpan 4.0 server (http://www.cbs.dtu.dk/services/NetMHCIIpan/) [101]. The MHC class II T cell epitopes were predicted for the eight common human alleles HLA-DRB1*01:01, -DRB1*03:01, -DRB1*04:01, -DRB1*07:01, -DRB1*08:01, -DRB1*11:01, -DRB1*13:01 and -DRB1*15:01 [102], and also the mouse alleles H-2-IAb, H-2-IAd and H-2-IEd. In IEDB, the prediction of MHC-II epitopes is performed by default in a set of 15-mer peptides overlapping by 10 amino acids and the peptides with lower percentile rank indicate higher affinity. NetMHCIIpan 4.0 informs if a sequence is a strong binding peptide (SB) or weak binding peptide (WB) based on %Rank < 1.0 or < 5, respectively, using artificial neural network algorithm.

### Construction of final vaccine sequence

The high-scored B-cell epitopes identified by various servers were analyzed and scanned for an overlapping sequence with the highly immunogenic MHC class II epitopes. The selected peptides were linked to each other by GPGPG linkers which conserve the independent immunological activities of the subunits and facilitate epitope presentation [103]. In addition, the domain 4 of Ply which was selected as a potential adjuvant was linked at the N-terminal of above construct via EAAAK linker to increase the immunogenicity of the epitope vaccine. EAAAK linker is a helical linker that can separate this domain from the other regions of the vaccine, providing proper structure for binding to the immune receptor [85]. A His-tag was added to the C-terminal of the final construct for the easy identification and purification of the vaccine.

### Evaluation of properties of the vaccine sequence

After designing and construction of the multi-epitope vaccine by positioning the selected amino acid sequences linked with suitable linkers, the most important features of the construct were evaluated using different bioinformatics tools. The physical and chemical properties like amino acid composition, molecular weight, theoretical isoelectric point, in vitro/in vivo half-life, instability index, aliphatic index and grand average of hydropathicity of the designed sequence were computed using the ExPASy ProtParam tool (http://web.expasy.org/protparam/) [82]. Solubility, antigenicity and allergenicity were evaluated for the final construct. SOLpro tool (https://scratch.proteomics.ics.uci.edu/) [104] implemented in the SCRATCH server, predicted the protein solubility on overexpression in *E. coli*. VaxiJen v2.0 (http://www.ddg-pharmfac.net/vaxijen/VaxiJen/VaxiJen.html) [105] and ANTIGENpro (http://scratch.proteomics.ics.uci.edu/) [106] were used to calculate the protein antigenicity. The VaxiJen is an alignment independent tool for antigenicity prediction with the default threshold of 0.4 and the accuracy of 70–89% according to target organism. The ANTIGENpro tool of SCRATCH server is an alignment-free and pathogen independent predictor for checking the protein antigenicity with the accuracy of 76% and threshold of 0.5. AlgPred (https://www.imtech.res.in/raghava/algpred/) [107] and AllerTOP (http://www.ddg-pharmfac.net/AllerTOP/) [108] were employed to predict allergenicity of the vaccine construct. Algpred uses various approaches consisting of SVM, motif-based and BLAST-search algorithms, and also hybrid approaches in order to allergenicity predictions and mapping of IgE peptides. AllerTOP, which is an alignment-independent server, predicts the allergens based on the physicochemical features of proteins.

### 3D structure modeling, refinement and validation of vaccine

The prediction of tertiary structure of the designed protein vaccine was done by the Robetta server (http://robetta.bakerlab.org/) [109]. This server can predict 3D models of proteins based either on comparative modeling or ab initio approaches. The GalaxyRefine server (http://galaxy.seoklab.org/cgi-bin/submit.cgi?type=REFINE) [92] was applied for the refinement of the best-modeled structure. The model validation was done through ERRAT [94], PROCHECK [93] and VERIFY-3D [110] at SAVES server (https://saves.mbi.ucla.edu/), and also ProSA-web server (https://prosa.services.came.sbg.ac.at/prosa.php) [111]. The VERIFY-3D is used to evaluate the compatibility of the 3D protein models with their own 1D amino acid sequences as measured by three-dimensional profiles. ProSA (Protein Structure Analysis) is a powerful tool for validation of the overall model quality which indicates whether the model has features characteristic for native structures.

### Interferon-gamma inducing peptide prediction

The peptides having the potential to activate IFN-γ inducing T-helper cells were predicted using online server IFNepitope (http://crdd.osdd.net/raghava/ifnepitope/) [112]. This server utilizes various approaches like motif-based method, machine learning algorithm, and hybrid approach. The prediction is based on a main dataset composed of 3705 IFN-gamma inducing and 6728 non-inducing MHC-II binders.

### Conformational B‑cell epitope prediction in the constructed vaccine

Conformational epitopes are comprised of residues that form the 3D structures recognized by the antibodies and play a key role in the humoral immunity. Therefore, the designed construct should have effective structural epitopes within its protein domains to provide more potent immunity. ElliPro (http://tools.iedb.org/ellipro/) [98] was used to identify the conformational/discontinuous B-cell epitopes in the refined final model of the multi-epitope vaccine by keeping the default parameters. Among the current tools for conformational epitope prediction, ElliPro is one of the best, with the remarkable AUC value of 0.732.

### Molecular docking between vaccine candidate and TLR-4

Protein–protein docking was performed through ClusPro web server (https://cluspro.org/login.php) [113] to determine the binding affinity of the multi-epitope vaccine and the toll-like receptor 4. The refined model of the vaccine as the ligand and the crystal structure of TLR4 (PDB ID: 3FXI) retrieved from RCSB (www.rcsb.org) [114] as the receptor were submitted to the server. The ClusPro applies three consecutive stages for docking process: (1) rigid-body docking using the fast Fourier transform correlation method, (2) clustering of the lowest-energy conformations, and (3) refinement of chosen conformations by energy minimization. The docked molecule was visualized by Discovery Studio Visualizer. The PDBsum tool available at http://www.ebi.ac.uk/thornton-srv/databases/pdbsum/Generate.html [115] was used to provide a graphical view of residual interaction between the vaccine and the receptor.

### Molecular dynamics simulation

The MD simulation was performed to confirm the structural stability of developed vaccine-TLR4 complex by the GROMACS 4.6.5 version [116] using the GROMOSE 54A7 force field [117], such that the applied conditions were similar to our previous studies [118, 119]. Briefly, the complex was positioned in a cubic box and solvated with TIP3P water model. The system neutralization was performed by the Na^+^ and Cl^−^ ions, followed by system energy minimization using the steepest descent algorithm. The system equilibration was done under 100 ps NVT at 300 K using Berendsen thermostat algorithm, followed by 100 ps NPT at 300 K and 1 bar using Parrinello Rahman barostat. The root mean square deviation (RMSD) versus time of the vaccine-TLR4 complex was plotted for 20 ns simulation to evaluate the system stability. The root mean square fluctuation (RMSF) per residue plots of the developed vaccine and the receptor were generated to verify the flexibility of the backbone atoms.

### Codon optimization and in silico cloning of the designed construct

The codon adaptation approach is applied to improve the expression of recombinant proteins. The vaccine protein sequence was submitted to the Java Codon Adaptation Tool (JCat) (http://www.jcat.de/) [120] for reverse translation and codon optimization according to the codon usage of *E. coli* (strain K12) as the expression host. The additional criteria of JCat were selected such as the avoidance of unwanted sites for Rho-independent transcription termination, prokaryotic ribosome binding and restriction enzymes. Finally, the optimized vaccine sequence, with restriction sites *Nde*I and *Xho*I added to the N- and C-terminus, respectively, was cloned in pET-28a(+) plasmid vector using the SnapGene tool (https://www.snapgene.com/try-snapgene/) to confirm the expression of the vaccine.

## Supplementary Information

**Additional file 1**.** Table S1**. Protein sequences of PspA1-5 (clades 1 to 5), PhtD and Ply. A region of PspA clade 2, B and C region of PspA clades 1 to 5 are underlined and shown in blue, red and green color, respectively. The C-terminal of PhtD (amino acid 383 to 853) and domain 4 of Ply (amino acid 360 to 471) are shown in color and underlined. In Ply4, the amino acids D385, C428 and W433, which must be replaced with N, G and F, respectively, are presented in blue color. Signal peptide is represented in lowercase italics. **Table S2**. The predicted B cell epitopes of A region of PspA2. **Table S3**. The predicted B cell epitopes of B region of PspA1. **Table S4**. The predicted B cell epitopes of B region of PspA2. **Table S5**. The predicted B cell epitopes of B region of PspA3. **Table S6**. The predicted B cell epitopes of B region of PspA4. **Table S7**. The predicted B cell epitopes of B region of PspA5. **Table S8**. The experimentally verified B cell epitopes from C region of PspAs. **Table S9**. The predicted linear B cell epitopes of PhtD-C. **Table S10**. The predicted discontinuous B cell epitopes of PhtD-C. **Table S11**. Prediction of MHC-II epitopes of PspA2-A and PspA1-5-B. The peptides with IEDB percentile rank <10.0 and NetMHCIIpan rank value <1.0 were considered for the next analysis. **Table S12**. Prediction of MHC-II epitopes of PhtD-C. The epitopes with IEDB percentile rank <10.0 and NetMHCIIpan rank value <1.0 were considered for the further analysis. **Figure S1**. Domain structure of PspA protein. Major domains of PspA are α-helical charged domain (amino acids 1-288) consisting of A, Aˊ and B regions, proline-rich domain (amino acids 289-370, C region), and choline-binding domain (amino acids 371-571). Within the α-HD, region B is a clade-defining region of the PspA molecule, which is represented by the stippled box. **Figure S2**. The results of transmembrane helices prediction. A and B show the prediction results of transmembrane helices in PspA2 and PhtD-C, respectively. The pink lines indicate the domains facing outside. **Figure S3**. The 3D models of PspA2 and PhtD-C. The predicted and refined 3D structures were visualized by the Discovery Studio Visualizer. **Figure S4**. The validation of refined model of PspA2 (A) and PhtD-C (B) with PROCHECK and ERRAT. Ramachandran plot of the structure of PspA2 or PhtD-C represents 82.1% or 87.2% residues in favored regions, respectively. In the ERRAT plot, the overall quality factor of structure of PspA2 or PhtD-C is 88.69% or 85.23%, respectively. **Figure S5**. The 3D models of B regions of PspA1-5. The homology modeled structures of B regions of PspA clades 1 to 5 (B1 to 5, respectively) visualized by Discovery Studio Visualizer. **Figure S6**. Codon optimization results of the designed construct. Codon optimization was performed using JCAT and codons were adapted for efficient expression in* E.coli* K12. The CAI index and GC content of the optimized sequence were 0.99 and 53.27%, respectively.

## Data Availability

All the data supporting the findings are contained within the manuscript.
