# Supplementary material for: Immunoinformatics-aided design of a new multi-epitope vaccine adjuvanted with domain 4 of pneumolysin against Streptococcus pneumoniae strains
Source: BMC Bioinformatics. 2023 Feb 24;24:67. doi: 10.1186/s12859-023-05175-6 (PMC9951839; doi:10.1186/s12859-023-05175-6)
Supplement: Supplementary file 1 — Additional file 1. Table S1. Protein sequences of PspA1-5 (clades 1 to 5), PhtD and Ply. A region of PspA clade 2, B and C region of PspA clades 1 to 5 are underlined and shown in blue, red and green color, respectively. The C-terminal of PhtD (amino acid 383 to 853) and domain 4 of Ply (amino acid 360 to 471) are shown in color and underlined. In Ply4, the amino acids D385, C428 and W433, which must be replaced with N, G and F, respectively, are presented in blue color. Signal peptide is represented in lowercase italics. Table S2. The predicted B cell epitopes of A region of PspA2. Table S3. The predicted B cell epitopes of B region of PspA1. Table S4. The predicted B cell epitopes of B region of PspA2. Table S5. The predicted B cell epitopes of B region of PspA3. Table S6. The predicted B cell epitopes of B region of PspA4. Table S7. The predicted B cell epitopes of B region of PspA5. Table S8. The experimentally verified B cell epitopes from C region of PspAs. Table S9. The predicted linear B cell epitopes of PhtD-C. Table S10. The predicted discontinuous B cell epitopes of PhtD-C. Table S11. Prediction of MHC-II epitopes of PspA2-A and PspA1-5-B. The peptides with IEDB percentile rank <10.0 and NetMHCIIpan rank value <1.0 were considered for the next analysis. Table S12. Prediction of MHC-II epitopes of PhtD-C. The epitopes with IEDB percentile rank <10.0 and NetMHCIIpan rank value <1.0 were considered for the further analysis. Figure S1. Domain structure of PspA protein. Major domains of PspA are α-helical charged domain (amino acids 1-288) consisting of A, Aˊ and B regions, proline-rich domain (amino acids 289-370, C region), and choline-binding domain (amino acids 371-571). Within the α-HD, region B is a clade-defining region of the PspA molecule, which is represented by the stippled box. Figure S2. The results of transmembrane helices prediction. A and B show the prediction results of transmembrane helices in PspA2 and PhtD-C, respectively. The pink lines in [file 12859_2023_5175_MOESM1_ESM.docx]

**Supplementary Tables and Figures.**

**Supplementary Table S1. Protein sequences of PspA1-5 (clades 1 to 5), PhtD and Ply.** A region of PspA clade 2, B and C region of PspA clades 1 to 5 are underlined and shown in blue, red and green color, respectively. The C-terminal of PhtD (amino acid 383 to 853) and domain 4 of Ply (amino acid 360 to 471) are shown in color and underlined. In Ply4, the amino acids D385, C428 and W433, which must be replaced with N, G and F, respectively, are presented in blue color. Signal peptide is represented in lowercase italics.

| Protein name | Strain | GenBank ID | Sequence |
| --- | --- | --- | --- |
| PspA1 | DBL6A | AAF27701.1 | *mnkkkmiltslasvailgaglvaspptvvra*EEAPVASQSKAEKDYDTAKRDAENAKKALEEAKRAQEKYADYQRRIEEKAAKETHASLEQQEANKDYQLKLKKYLDGRNLSNSSVLKKEMEEAEKKDKEKQAEFNKIRREIVVPNPQELEMARRKSEVAKPKESGLVKRVEEAEKKVTEARPKLDAERAKEVVLQAQIAELENEVHKLEPKLKEIDESDSEDYVKEGFRAPLQSELDAKQAKLSKLEELSDKIDELDAEIAKLEKDVEDFKNSDGEQAGQYLAAAEEDLIAKKAELEQTEADLKKAVNEPEKPAPAPAPETPAPEAPAEQPKPAPETPAPAPKPEKPAEQPKPEKPADQQAEEDYARRSEEEYNRLTQQQPAPAPKPEQPAKPEKPAEEPTQPEK |
| PspA2 | WU2 | AAF27710.1 | *mnkkkmiltslasvailgaglvasqptlvra*EESPVASQSKAEKDYDAAVKKSEAAKKAYEEAKKALEEAKVAQKKYEDDQKKTEEKAELEKEASEAIAKATEEVQQAYLAYQRASNKAEAAKMIEEAQRRENEARAKFTTIRTTMVVPEPEQLAETKKKAEEAKAKEPKLAKKAAEAKAKLEEAEKKATEAKPQVDAEEVAPQAKIAELENQVHRLEQELKEIDESESEDYAKEGFRAPLQSKLDAKKAKLSKLEELSDKIDELDAEIAKLEDQLKAVEENNNVEDYSTEGLEKTIAAKKTELEKTEADLKKAVNEPEKSAEEPSQPEKPAEEAPAPEQPTEPTQPEKPAEETPAPKPEKPAEQPNAEKTDDQQAEEDYARRSEEEYNRLTQQQPPKAEKPAPAPQPEQTSSLH |
| PspA3 | BG8090 | AAF27713.1 | *mnkkkmiltslasvailgagfvtsqptfvra*EEAPQVVEKSSLEKKYEEAKAKADTAKKDYETAKKKAEDAQKKYDEDQKKTEDKAKAVKKVDEELQKANLELQKAHVKYQKAQRELSESPDRRKSAARKKLADALSYIGEVELKQKEAEANFNTEQAKVIPKETELAVTKQKAEEAKKEAEVAKKKFDKAAQEVEVAKKEVEAKELEIEKLQDEISTLEQEVATAQHQVDNLKKLLAGVDPDDTEAIEAKLKKGEAELNAKQAELAKKQTELEKLLDSLDPEGKTQDELDKEAAEAELNKKVESLQNKVADLEKEISNLEILLGGADSEDDTAALQNKLAAKQAELAKKQTELEKLLDNLDPEGKTQDELDKEAAEAELDKKADELQNKVADLEKEISNLEILLGGADPEDDTAALQNKLATKKAEFEKTQKELDAALNELGPDGDEEETPAPAPAPKPEQPAPAPAPKPEQPAPAPAPKPEQPAPAPAPKPEQPTPAPKS |
| PspA4 | EF5668 | AAC62252.1 | *mnkkkmiltslasvailgagfvassptfvra*EEAPVANQSKAEKDYDAAVKKSEAAKKDYETAKKKAEDAQKKYDEDQKKTEAKAEKERKASEKIAEATKEVQQAYLAYLQASNESQRKEADKKIKEATQRKDEAEAAFATIRTTIVVPEPSELAETKKKAEEATKEAEVAKKKSEEAAKEVEVEKNKILEQDAENEKKIDVLQNKVADLEKGIAPYQNEVAELNKEIARLQSDLKDAEENNVEDYIKEGLEQAITNKKAELATTQQNIDKTQKDLEDAELELEKVLATLDPEGKTQDELDKEAAEAELNEKVEALQNQVAELEEELSKLEDNLKDAETNNVEDYIKEGLEEAIATKKAELEKTQKELDAALNELGPDGDEEETPAPAPQPEKPAEEPENPAPAPKPEKSADQQAEEDYARRSEEEYNRLTQQQPPKAEKPAPAPQPEQPAPAPKIGWKQENGMWYFYNTDGSMATGWLQNNGSWYYLNSNGAMATGWLQYNGSWYYLNANGAMATGWLQYNGSWYYLNANGAMATGWLQYNGSWYYLNANGDMATGWLQYNGSWYYLNANGDMATGWAKVHGSWYYLNANGSMATGWVKDGETWYYLEASGSMKANQWFQVSDKWYYVNGLGSLSVNTTVDGYKVNANGEWV |
| PspA5 | ATCC6303 | AAF27715.1 | *mnkkkmiltslasvailgtgfvassptfvra*EESPQVVEKSSLEKKYEEAKAKADTAKKDYETAKKKAEDAQKKYDEDQKKTEDKAKAVKKVDEERQKANLAVQKAYVEYREAKDKASAEKKIEEAKRKQKEANKKFNEEQAKVVPEAKELAATKQKAEKAKKDAEVAKEKYDKAVQEVEVEKNKILEQDAENEKKIDVLQNKVADLEKGIAPYQNKVAELNKEIARLQSDLKDAEENNVEDYIKEGLEQAIADKKAELATTQQNIDKTQKDLEDAELELEKVLATLDPEGKTQDELDKEAAEDANIEALQNKVADLENKVAELDKEVTRLQSDLKDAEENNVEDYVKEGLEKALTDKKVELNNTQKALDTAPKALDTALNELGPDGDEEETPAPAPKPEQPAEQPKPAPAPKPEKTDDQQAEEDYARRSEEEYNRLPQQQPPKAEKPAPAPKPEQPVPAP |
| PhtD | R6 | AAK99711.1 | *mkinkkylagsvavlalsv*CSYELGRHQAGQVKKESNRVSYIDGDQAGQKAENLTPDEVSKREGINAEQIVIKITDQGYVTSHGDHYHYYNGKVPYDAIISEELLMKDPNYQLKDSDIVNEIKGGYVIKVDGKYYVYLKDAAHADNIRTKEEIKRQKQERSHNHNSRADNAVAAARAQGRYTTDDGYIFNASDIIEDTGDAYIVPHGDHYHYIPKSDLSASELAAAQAYWNGKQGSRPSSSSSHNANPAQPRLSENHNLTVTPTYHQNQGENISSLLRELYAKPLSERHVESDGLIFDPAQITSRTANGVAVPHGDHYHFIPYSQLSPLEEKLARIIPLRYRSNHWVPDSRPEQPSPQSTPEPSPSPQPAPNPQPAPSNPIDEKLVKEAVRKVGDGYVFEENGVPRYIPAKDLSAETAAGIDSKLAKQESLSHKLGAKKTDLPSSDREFYNKAYDLLARIHQDLLDNKGRQVDFEALDNLLERLKDVSSDKVKLVDDILAFLAPIRHPERLGKPNAQITYTDDEIQVAKLAGKYTTEDGYIFDPRDITSDEGDAYVTPHMTHSHWIKKDSLSEAERAAAQAYAKEKGLTPPSTDHQDSGNTEAKGAEAIYNRVKAAKKVPLDRMPYNLQYTVEVKNGSLIIPHYDHYHNIKFEWFDEGLYEAPKGYSLEDLLATVKYYVEHPNERPHSDNGFGNASDHVQRNKNGQADTNQTEKPNEEKPQTEKPEEDKEHDEVSEPTHPESDEKENHVGLNPSADNLYKPSTDTEETEEEAEDTTDEAEIPQVEHSVINAKIAEAEALLEKVTDSSIRQNAVETLTGLKSSLLLGTKDNNTISAEVDSLLALLKESQPTPIQ |
| Ply | D39 | ABJ53672.1 | MANKAVNDFILAMNYDKKKLLTHQGESIENRFIKEGNQLPDEFVVIERKKRSLSTNTSDISVTATNDSRLYPGALLVVDETLLENNPTLLAVDRAPMTYSIDLPGLASSDSFLQVEDPSNSSVRGAVNDLLAKWHQDYGQVNNVPARMQYEKITAHSMEQLKVKFGSDFEKTGNSLDIDFNSVHSGEKQIQIVNFKQIYYTVSVDAVKNPGDVFQDTVTVEDLKQRGISAERPLVYISSVAYGRQVYLKLETTSKSDEVEAAFEALIKGVKVAPQTEWKQILDNTEVKAVILGGDPSSGARVVTGKVDMVEDLIQEGSRFTADHPGLPISYTTSFLRDNVVATFQNSTDYVETKVTAYRNGDLLLDHSGAYVAQYYITWDELSYDHQGKEVLTPKAWDRNGQDLTAHFTTSIPLKGNVRNLSVKIRECTGLAWEWWRTVYEKTDLPLVRKRTISIWGTTLYPQVEDKVEND |

**Supplementary Table S2. The predicted B cell epitopes of A region of PspA2.**

| LBTope* | | | | | | | | |
| --- | --- | --- | --- | --- | --- | --- | --- | --- |
| Sequence | **% Probabilty of correct prediction** | **Sequence** | | **% Probabilty of correct prediction** | **Sequence** | **% Probabilty of correct prediction** | **Sequence** | **% Probabilty of correct prediction** |
| SEAAKKAYEEAKKAL | 62.31 | KKALEEAKVAQKKYE | | 76.33 | EAKVAQKKYEDDQKK | 83.58 | QKKYEDDQKKTEEKA | 67.04 |
| EAAKKAYEEAKKALE | 69.60 | KALEEAKVAQKKYED | | 84.87 | AKVAQKKYEDDQKKT | 74.88 | KKYEDDQKKTEEKAE | 68.92 |
| EEAKKALEEAKVAQK | 64.08 | ALEEAKVAQKKYEDD | | 82.04 | KVAQKKYEDDQKKTE | 71.00 | KYEDDQKKTEEKAEL | 71.27 |
| EAKKALEEAKVAQKK | 71.41 | LEEAKVAQKKYEDDQ | | 84.93 | VAQKKYEDDQKKTEE | 86.43 | YEDDQKKTEEKAELE | 68.04 |
| AKKALEEAKVAQKKY | 66.51 | EEAKVAQKKYEDDQK | | 78.49 | AQKKYEDDQKKTEEK | 77.81 | KAEAAKMIEEAQRR | 63.62 |
| ABCpred | | | | | | | | |
| Sequence | **Score** | **Sequence** | | **Score** | **Sequence** | **Score** | **Sequence** | **Score** |
| AKVAQKKYEDDQKKTE | 0.93 | EEVQQAYLAYQRASNK | | 0.84 | YEDDQKKTEEKAELEK | 0.79 | YQRASNKAEAAKMIEE | 0.57 |
| AELEKEASEAIAKATE | 0.92 | SEAAKKAYEEAKKALE | | 0.83 | EKDYDAAVKKSEAAKK | 0.73 |  |  |
| YEEAKKALEEAKVAQK | 0.89 | PVASQSKAEKDYDAAV | | 0.82 | SEAIAKATEEVQQAYL | 0.70 |  |  |
| Emini surface accessibility Prediction | | | | | | | | |
| Sequence | | **Sequence** | | |  | |  | |
| KKYEDDQKKTEE | | SKAEKD | | |  | |  | |
|  |  |  | |  |  |  |  |  |
| Ellipro (predicted linear epitopes) | | | | | | | | |
| Sequence | **Score** | | **Sequence** | **Score** | **Sequence** | **Score** |  |  |
| EESPVASQSKAEKDYDA | 0.806 | | LEKEASEAIAKATEEVQQA | 0.63 | RASNKAE | 0.563 |  |  |
| Ellipro (predicted discontinuous epitopes) | | | | | | | | |
| Residues | **Score** | | **Residues** | **Score** | **Residues** | **Score** | **Residues** | **Score** |
| E1, E2, S3, P4, V5, A6, S7, Q8, S9, K10, A11, E12, K13, D14, D16, A17 | 0.828 | | L59, E60, E62, A63, S64, E65, A66, I67, A68, K169, A70, T71, E72, E73, V74, Q76, A77, A80, R83 | 0.675 | K44, K45, D48, D49, K51, K52, E55 | 0.671 | A84, K87, A88, E89 | 0.585 |
| DiscoTope 2.0 | | | | | | | | |
| Sequence |  | | **Sequence** |  | **Sequence** |  |  |  |
| EESPVASQSKAEKDYDAAVKKSEAAKK | | | KKALEEAKVAQKKYEDDQKKTEEKAELE | | VQQAYLAYQRASNKAEAAKMIEEAQRR | |  |  |
| *LBtope assigns scores between 0-100% to each of the identified epitopes. In this study, only those epitopes with the scores above 60% are considered. | | | | | | | | |

**Supplementary Table S3. The predicted B cell epitopes of B region of PspA1.**

| LBTope* | | | | | | | | |
| --- | --- | --- | --- | --- | --- | --- | --- | --- |
| Sequence | **% Probabilty of correct prediction** | **Sequence** | | **% Probabilty of correct prediction** | **Sequence** | **% Probabilty of correct prediction** | **Sequence** | **% Probabilty of correct prediction** |
| KEIDESDSEDYVKEG | 64.42 | SKLEELSDKIDELDA | | 73.12 | DAEIAKLEKDVEDFK | 85.79 | QAGQYLAAAEEDLIA | 60.75 |
| EIDESDSEDYVKEGF | 60.99 | KLEELSDKIDELDAE | | 64.43 | AEIAKLEKDVEDFKN | 90.68 | AGQYLAAAEEDLIAK | 71.84 |
| IDESDSEDYVKEGFR | 62.63 | LEELSDKIDELDAEI | | 72.47 | EIAKLEKDVEDFKNS | 88.37 | GQYLAAAEEDLIAKK | 73.32 |
| DESDSEDYVKEGFRA | 69.16 | EELSDKIDELDAEIA | | 73.55 | IAKLEKDVEDFKNSD | 70.88 | QYLAAAEEDLIAKKA | 64.78 |
| ESDSEDYVKEGFRAP | 65.61 | ELSDKIDELDAEIAK | | 66.99 | AKLEKDVEDFKNSDG | 63.89 | YLAAAEEDLIAKKAE | 60.07 |
| SEDYVKEGFRAPLQS | 60.02 | LSDKIDELDAEIAKL | | 72.94 | KLEKDVEDFKNSDGE | 61.30 | LAAAEEDLIAKKAEL | 66.52 |
| EDYVKEGFRAPLQSE | 60.02 | SDKIDELDAEIAKLE | | 73.56 | LEKDVEDFKNSDGEQ | 62.15 | AAAEEDLIAKKAELE | 66.56 |
| DYVKEGFRAPLQSEL | 75.49 | DKIDELDAEIAKLEK | | 89.09 | DVEDFKNSDGEQAGQ | 73.90 | AAEEDLIAKKAELEQ | 61.32 |
| YVKEGFRAPLQSELD | 66.81 | KIDELDAEIAKLEKD | | 81.34 | EDFKNSDGEQAGQYL | 64.85 | LEQTEADLKKAVNEX | 65.55 |
| VKEGFRAPLQSELDA | 60.23 | IDELDAEIAKLEKDV | | 84.23 | DFKNSDGEQAGQYLA | 60.23 | EQTEADLKKAVNEXX | 72.01 |
| EGFRAPLQSELDAKQ | 65.46 | DELDAEIAKLEKDVE | | 84.26 | FKNSDGEQAGQYLAA | 66.00 | QTEADLKKAVNEXXX | 66.31 |
| ELDAKQAKLSKLEEL | 65.31 | ELDAEIAKLEKDVED | | 94.31 | DGEQAGQYLAAAEED | 72.47 | TEADLKKAVNEXXXX | 67.83 |
| LDAKQAKLSKLEELS | 62.05 | LDAEIAKLEKDVEDF | | 81.43 | GEQAGQYLAAAEEDL | 65.13 | EADLKKAVNEXXXXX | 60.02 |
| ABCpred | | | | | | | | |
| Sequence | **Score** | **Sequence** | | **Score** | **Sequence** | **Score** | **Sequence** | **Score** |
| LKEIDESDSEDYVKEG | 0.86 | DSEDYVKEGFRAPLQS | | 0.83 | LDAEIAKLEKDVEDFK | 0.80 | AELEQTEADLKKAVNE | 0.75 |
| SDKIDELDAEIAKLEK | 0.85 | AGQYLAAAEEDLIAKK | | 0.80 | APLQSELDAKQAKLSK | 0.78 |  |  |
| Emini surface accessibility Prediction | | | | | | | | |
| Sequence | | **Sequence** | | | **Sequence** | | **Sequence** | |
| LKEIDESDSEDYVKEG | | SELDAKQAKLSK | | | EELSDKIDELDAE | | AKLEKDVEDFKNSDGEQA | |
|  |  |  | |  |  |  |  |  |
| Ellipro (predicted linear epitopes) | | | | | | | | |
| Sequence | **Score** | | **Sequence** | **Score** | **Sequence** | **Score** |  |  |
| LKEIDESDSEDYVKEGFRAP | 0.755 | | KDVEDFKNSDGEQAGQYLA | 0.698 | ADLKKAVNE | 0.656 |  |  |
| Ellipro (predicted discontinuous epitopes) | | | | | | | | |
| Residues | **Score** | | **Residues** | **Score** | **Residues** | **Score** | **Residues** | **Score** |
| L1, K2, E3, I4, D5, E6, S7, D8, S9, E10, D11, Y12, V13, K14, E15, G16, F17, R18, A19, P20 | 0.755 | | D58, F59, K60, N61, S62, D63, G64, E65, Q66, A67, G68, Q69, Y70, A72, E76 | 0.747 | A83, E86, Q87, A90, D91, K93 | 0.643 | K54, D55, E57 | 0.616 |
| DiscoTope 2.0 | | | | | | | | |
| Sequence |  | | **Sequence** |  |  |  |  |  |
| LKEIDESDSEDYVKEGFRAPLQS | | | KNSDGEQAGQ |  |  |  |  |  |
|  | | |  |  |  |  |  |  |
| *LBtope assigns scores between 0-100% to each of the identified epitopes. In this study, only those epitopes with the scores above 60% are considered. | | | | | | | | |

**Supplementary Table S4. The predicted B cell epitopes of B region of PspA2.**

| LBTope* | | | | | | | | |
| --- | --- | --- | --- | --- | --- | --- | --- | --- |
| Sequence | **% Probabilty of correct prediction** | **Sequence** | | **% Probabilty of correct prediction** | **Sequence** | **% Probabilty of correct prediction** | **Sequence** | **% Probabilty of correct prediction** |
| SEDYAKEGFRAPLQS | 61.34 | QSKLDAKKAKLSKLE | | 61.90 | SDKIDELDAEIAKLE | 73.56 | KTEADLKKAVNEPEK | 66.71 |
| EDYAKEGFRAPLQSK | 66.76 | KLDAKKAKLSKLEEL | | 61.47 | DKIDELDAEIAKLED | 87.06 | TEADLKKAVNEPEKS | 77.04 |
| DYAKEGFRAPLQSKL | 72.16 | LDAKKAKLSKLEELS | | 72.99 | KIDELDAEIAKLEDQ | 70.13 | EADLKKAVNEPEKSA | 84.97 |
| YAKEGFRAPLQSKLD | 72.65 | DAKKAKLSKLEELSD | | 62.71 | IDELDAEIAKLEDQL | 60.87 | ADLKKAVNEPEKSAE | 86.57 |
| AKEGFRAPLQSKLDA | 60.75 | AKKAKLSKLEELSDK | | 62.45 | AEIAKLEDQLKAVEE | 66.98 | DLKKAVNEPEKSAEE | 87.42 |
| KEGFRAPLQSKLDAK | 60.75 | KKAKLSKLEELSDKI | | 60.51 | EIAKLEDQLKAVEEN | 66.02 | LKKAVNEPEKSAEEP | 71.72 |
| EGFRAPLQSKLDAKK | 73.04 | SKLEELSDKIDELDA | | 73.12 | IAKLEDQLKAVEENN | 60.01 | KKAVNEPEKSAEEPS | 69.75 |
| GFRAPLQSKLDAKKA | 61.68 | KLEELSDKIDELDAE | | 64.43 | VEENNNVEDYSTEGL | 69.65 | KAVNEPEKSAEEPSQ | 70.07 |
| FRAPLQSKLDAKKAK | 70.16 | LEELSDKIDELDAEI | | 72.47 | EENNNVEDYSTEGLE | 68.29 | AVNEPEKSAEEPSQ | 66.04 |
| RAPLQSKLDAKKAKL | 62.13 | EELSDKIDELDAEIA | | 73.55 | ENNNVEDYSTEGLEK | 62.45 |  |  |
| APLQSKLDAKKAKLS | 69.39 | ELSDKIDELDAEIAK | | 66.99 | NNNVEDYSTEGLEKT | 61.12 |  |  |
| PLQSKLDAKKAKLSK | 71.28 | LSDKIDELDAEIAKL | | 72.94 | EKTEADLKKAVNEPE | 66.71 |  |  |
| ABCpred | | | | | | | | |
| Sequence | **Score** | **Sequence** | | **Score** | **Sequence** | **Score** | **Sequence** | **Score** |
| ESEDYAKEGFRAPLQS | 0.90 | LKEIDESESEDYAKEG | | 0.81 | NNNVEDYSTEGLEKTI | 0.75 | TIAAKKTELEKTEADL | 0.68 |
| SDKIDELDAEIAKLED | 0.87 | ADLKKAVNEPEKSAEE | | 0.79 | LEDQLKAVEENNNVED | 0.73 |  |  |
| ELEKTEADLKKAVNEP | 0.83 | YSTEGLEKTIAAKKTE | | 0.78 | APLQSKLDAKKAKLSK | 0.70 |  |  |
| Emini surface accessibility Prediction | | | | | | | | |
| Sequence | | **Sequence** | | | **Sequence** | |  | |
| SESEDYA | | ENNNVE | | | KKTELEKT | |  | |
|  |  |  | |  |  |  |  |  |
| Ellipro (predicted linear epitopes) | | | | | | | | |
| Sequence | **Score** | | **Sequence** | **Score** | **Sequence** | **Score** |  |  |
| LKEIDESESEDYAKEGFR | 0.771 | | KAVEENNNVEDYSTE | 0.755 | KKAVNE | 0.739 |  |  |
| Ellipro (predicted discontinuous epitopes) | | | | | | | | |
| Residues | **Score** | | **Residues** | **Score** | **Residues** | **Score** | **Residues** | **Score** |
| K57, A58, V59, E60, E61, N62, N63, N64, V65, E66 | 0.826 | | L1, K2, E3, I4, D5, E6, S7, E8, D11, Y12, A13, K14, E15, G16, F17, R18 | 0.749 | Y68, S69, E71, K75 | 0.644 | T82, E85, K86 | 0.636 |
| DiscoTope 2.0 | | | | | | | | |
| Sequence |  | | **Sequence** |  |  |  |  |  |
| LKEIDESESEDYAKEGFRAPLQSKLDAKKA | | | VEENNNVEDYS |  |  |  |  |  |
| *LBtope assigns scores between 0-100% to each of the identified epitopes. In this study, only those epitopes with the scores above 60% are considered. | | | | | | | | |

**Supplementary Table S5. The predicted B cell epitopes of B region of PspA3.**

| LBTope* | | | | | | | | |
| --- | --- | --- | --- | --- | --- | --- | --- | --- |
| Sequence | **% Probabilty of correct prediction** | **Sequence** | | **% Probabilty of correct prediction** | **Sequence** | **% Probabilty of correct prediction** | **Sequence** | **% Probabilty of correct prediction** |
| LAKKQTELEKLLDN | 64.92 | KLLDNLDPEGKTQDE | | 61.67 | AAEAELDKKADELQN | 62.11 | LLGGADPEDDTAALQ | 72.06 |
| LAKKQTELEKLLDNL | 74.17 | LLDNLDPEGKTQDEL | | 64.08 | ADELQNKVADLEKEI | 61.30 | LGGADPEDDTAALQN | 67.89 |
| AKKQTELEKLLDNLD | 77.67 | DPEGKTQDELDKEAA | | 61.55 | DELQNKVADLEKEIS | 64.94 | GGADPEDDTAALQNK | 70.79 |
| KKQTELEKLLDNLDP | 67.79 | DELDKEAAEAELDKK | | 63.99 | LQNKVADLEKEISNL | 64.40 | GADPEDDTAALQNKL | 64.04 |
| KQTELEKLLDNLDPE | 63.37 | ELDKEAAEAELDKKA | | 69.96 | VADLEKEISNLEILL | 63.63 | ADPEDDTAALQNKLA | 65.84 |
| TELEKLLDNLDPEGK | 66.87 | LDKEAAEAELDKKAD | | 75.10 | EKEISNLEILLGGAD | 60.47 | DPEDDTAALQNKLAT | 67.05 |
| ELEKLLDNLDPEGKT | 66.58 | DKEAAEAELDKKADE | | 76.94 | EISNLEILLGGADPE | 60.66 |  |  |
| LEKLLDNLDPEGKTQ | 67.95 | KEAAEAELDKKADEL | | 66.89 | ISNLEILLGGADPED | 71.86 |  |  |
| EKLLDNLDPEGKTQD | 69.42 | EAAEAELDKKADELQ | | 60.35 | SNLEILLGGADPEDD | 65.61 |  |  |
| ABCpred | | | | | | | | |
| Sequence | **Score** | **Sequence** | | **Score** | **Sequence** | **Score** | **Sequence** | **Score** |
| AEFEKTQKELDAALNE | 0.89 | ILLGGADPEDDTAALQ | | 0.80 | NKLATKKAEFEKTQKE | 0.71 | EKLLDNLDPEGKTQDE | 0.70 |
| DPEGKTQDELDKEAAE | 0.89 | DPEDDTAALQNKLATK | | 0.75 | AAEAELDKKADELQNK | 0.71 | AKKQTELEKLLDNLDP | 0.64 |
| Emini surface accessibility Prediction | | | | | | | | |
| Sequence | | **Sequence** | | | **Sequence** | |  | |
| EGKTQDELD | | ELDKKAD | | | EKTQKE | |  | |
|  |  |  | |  |  |  |  |  |
| Ellipro (predicted linear epitopes) | | | | | | | | |
| Sequence | **Score** | | **Sequence** | **Score** | **Sequence** | **Score** |  |  |
| SNLEILLGGADPEDD | 0.718 | | DAALNE | 0.69 | TELEKLLDNLDPEGKTQDEL | 0.674 |  |  |
| Ellipro (predicted discontinuous epitopes) | | | | | | | | |
| Residues | **Score** | | **Residues** | **Score** | **Residues** | **Score** | **Residues** | **Score** |
| K10, L11, L12, D13, N14, L15, D16, P17, E18, G19, K20, T21, Q22, D23, E24, L25 | 0.719 | | S53, N54, L55, E56, I57, L58, L59, G60, G61, A62, D63, P64, E65, D66, D67 | 0.718 | D90, A91, A92, L93, N94, E95 | 0.69 | A80, E83, K84 | 0.656 |
| DiscoTope 2.0 | | | | | | | | |
| Sequence |  | |  |  |  |  |  |  |
| AKKQTELEKLLDNLDPEGKTQDE | | |  |  |  |  |  |  |
| *LBtope assigns scores between 0-100% to each of the identified epitopes. In this study, only those epitopes with the scores above 60% are considered. | | | | | | | | |

**Supplementary Table S6. The predicted B cell epitopes of B region of PspA4.**

| LBTope* | | | | | | | | |
| --- | --- | --- | --- | --- | --- | --- | --- | --- |
| Sequence | **% Probabilty of correct prediction** | **Sequence** | | **% Probabilty of correct prediction** | **Sequence** | **% Probabilty of correct prediction** | **Sequence** | **% Probabilty of correct prediction** |
| DPEGKTQDELDKEAA | 61.55 | NQVAELEEELSKLED | | 65.74 | ELEEELSKLEDNLKD | 78.88 | EELSKLEDNLKDAET | 69.15 |
| NEKVEALQNQVAELE | 62.32 | QVAELEEELSKLEDN | | 64.53 | LEEELSKLEDNLKDA | 79.61 | ELSKLEDNLKDAETN | 63.01 |
| LQNQVAELEEELSKL | 62.89 | VAELEEELSKLEDNL | | 66.57 | EEELSKLEDNLKDAE | 78.33 | KLEDNLKDAETNNVE | 60.23 |
| ABCpred | | | | | | | | |
| Sequence | **Score** | **Sequence** | | **Score** | **Sequence** | **Score** |  |  |
| AELEKTQKELDAALNE | 0.95 | LEKVLATLDPEGKTQD | | 0.79 | ELSKLEDNLKDAETNN | 0.73 |  |  |
| DPEGKTQDELDKEAAE | 0.89 | EGLEEAIATKKAELEK | | 0.78 | EDAELELEKVLATLDP | 0.69 |  |  |
| VEDYIKEGLEEAIATK | 0.80 | QVAELEEELSKLEDNL | | 0.76 | EAAEAELNEKVEALQN | 0.63 |  |  |
| Emini surface accessibility Prediction | | | | | | | | |
| Sequence | | **Sequence** | | | **Sequence** | |  | |
| PEGKTQDELD | | NLKDAE | | | KKAELEKTQKE | |  | |
|  |  |  | |  |  |  |  |  |
| Ellipro (predicted linear epitopes) | | | | | | | | |
| Sequence | **Score** | | **Sequence** | **Score** |  |  |  |  |
| PEGKTQDELDKEAAEA | 0.758 | | DNLKDAETNNVEDYIKE | 0.749 |  |  |  |  |
| Ellipro (predicted discontinuous epitopes) | | | | | | | | |
| Residues | **Score** | | **Residues** | **Score** | **Residues** | **Score** | **Residues** | **Score** |
| D61, A62, E63, T64, N65, N66, V67, E68, D69, I71 | 0.828 | | P17, E18, G19, K20, T21, Q22, D23, E24, L25, D26, K27, E28, A29, A30, E31, A32 | 0.758 | D94, A95, L97, N98 | 0.722 | Y70, E73, E77 | 0.684 |
| DiscoTope 2.0 | | | | | | | | |
| Sequence |  | | **Sequence** |  |  |  |  |  |
| KTQDELDKE |  | | KDAETNNVEDYIKE |  |  |  |  |  |
| *LBtope assigns scores between 0-100% to each of the identified epitopes. In this study, only those epitopes with the scores above 60% are considered. | | | | | | | | |

**Supplementary Table S7. The predicted B cell epitopes of B region of PspA5.**

| LBTope* | | | | | | | | |
| --- | --- | --- | --- | --- | --- | --- | --- | --- |
| Sequence | **% Probabilty of correct prediction** | **Sequence** | | **% Probabilty of correct prediction** | **Sequence** | **% Probabilty of correct prediction** | **Sequence** | **% Probabilty of correct prediction** |
| DPEGKTQDELDKEAA | 61.55 | QDELDKEAAEDANIE | | 81.37 | LENKVAELDKEVTRL | 74.49 | ENNVEDYVKEGLEKA | 61.77 |
| EGKTQDELDKEAAED | 65.40 | DELDKEAAEDANIEA | | 81.10 | ENKVAELDKEVTRLQ | 74.06 | NNVEDYVKEGLEKAL | 60.38 |
| GKTQDELDKEAAEDA | 76.32 | ELDKEAAEDANIEAL | | 64.59 | NKVAELDKEVTRLQS | 63.68 | EDYVKEGLEKALTDK | 68.01 |
| KTQDELDKEAAEDAN | 72.02 | VADLENKVAELDKEV | | 67.54 | AEENNVEDYVKEGLE | 64.37 | DYVKEGLEKALTDKK | 67.86 |
| TQDELDKEAAEDANI | 79.69 | DLENKVAELDKEVTR | | 67.52 | EENNVEDYVKEGLEK | 62.80 |  |  |
| ABCpred | | | | | | | | |
| Sequence | **Score** | **Sequence** | | **Score** | **Sequence** | **Score** | **Sequence** | **Score** |
| DPEGKTQDELDKEAAE | 0.89 | EVTRLQSDLKDAEENN | | 0.81 | EDAELELEKVLATLDP | 0.69 | ADLENKVAELDKEVTR | 0.58 |
| EGLEKALTDKKVELNN | 0.84 | LEKVLATLDPEGKTQD | | 0.79 | ELDKEAAEDANIEALQ | 0.68 | EDANIEALQNKVADLE | 0.58 |
| KALDTAPKALDTALNE | 0.81 | DKKVELNNTQKALDTA | | 0.70 | VEDYVKEGLEKALTDK | 0.64 |  |  |
| Emini surface accessibility Prediction | | | | | | | | |
| Sequence | | **Sequence** | | | **Sequence** | |  | |
| PEGKTQDELD | | LKDAEENN | | | NNTQKA | |  | |
|  |  |  | |  |  |  |  |  |
| Ellipro (predicted linear epitopes) | | | | | | | | |
| Sequence | **Score** | | **Sequence** | **Score** | **Sequence** | **Score** |  |  |
| PEGKTQDELDKEAAEDANI | 0.76 | | SDLKDAEENNVEDYVKEG | 0.755 | QKALDTAPK | 0.678 |  |  |
| Ellipro (predicted discontinuous epitopes) | | | | | | | | |
| Residues | **Score** | | **Residues** | **Score** | **Residues** | **Score** |  |  |
| D65, A66, E67, E68, N69, N70, V71, E72, D73, Y74, V75, K76, E77, G78, K81 | 0.776 | | P17, E18, G19, K20, T21, Q22, D23, E24, L25, D26, K27, E28, A29, A30, E31, D32, A33, N34, I35 | 0.76 | K95, A96, L97, D98, T99, A100, P101, K102, D105, T106, L108, N109 | 0.696 |  |  |
| DiscoTope 2.0 | | | | | | | | |
| Sequence |  | | **Sequence** |  |  |  |  |  |
| KTQDELDKEAAEDAN |  | | KDAEENNVEDYVKEG |  |  |  |  |  |
| *LBtope assigns scores between 0-100% to each of the identified epitopes. In this study, only those epitopes with the scores above 60% are considered. | | | | | | | | |

**Supplementary Table S8. The experimentally verified B cell epitopes from C region of PspAs.**

| Experimentally approved epitopes of PspA-C | Ref. |
| --- | --- |
| EKSADQQAEEDYARRSEEEYNRLTQQQ | Daniels 2010 |
| PKPEQ |  |
| PAPAPKPEQPAPAPK | Vadesilho 2014 |
| APKPE |  |
| APKPEQPA |  |
| EEDYARRSEEEYNRL |  |
| PAPKPEQPAEQPKPAPAPQPAPAPKPEKT | Tamborrini 2015 |
| QQAEEDYARRSEEEYNRLPQQQPPKAEKP |  |
| PKPEQPAPAPK |  |
| PKPEQPAPAPKPEQPAKPEKP |  |
| PEQPAKPEKP |  |

**Supplementary Table S9. The predicted linear B cell epitopes of PhtD-C.**

| LBTope* | | | | | | | | |
| --- | --- | --- | --- | --- | --- | --- | --- | --- |
| Sequence | **% Probabilty of correct prediction** | **Sequence** | | **% Probabilty of correct prediction** | **Sequence** | **% Probabilty of correct prediction** | **Sequence** | **% Probabilty of correct prediction** |
| KVGDGYVFEENGVPR | 62.17 | APIRHPERLGKPNAQ | | 62.70 | FEWFDEGLYEAPKGY | 64.84 | EDKEHDEVSEPTHPE | 60.76 |
| VGDGYVFEENGVPRY | 62.30 | PIRHPERLGKPNAQI | | 60.66 | EWFDEGLYEAPKGYS | 65.38 | DKEHDEVSEPTHPES | 63.36 |
| GDGYVFEENGVPRYI | 68.29 | IFDPRDITSDEGDAY | | 61.34 | GLYEAPKGYSLEDLL | 62.65 | KEHDEVSEPTHPESD | 72.74 |
| DGYVFEENGVPRYIP | 74.69 | FDPRDITSDEGDAYV | | 65.07 | LYEAPKGYSLEDLLA | 71.19 | EHDEVSEPTHPESDE | 56.97 |
| GYVFEENGVPRYIPA | 62.80 | WIKKDSLSEAERAAA | | 66.66 | YEAPKGYSLEDLLAT | 61.84 | HDEVSEPTHPESDEK | 58.14 |
| PRYIPAKDLSAETAA | 61.01 | IKKDSLSEAERAAAQ | | 69.18 | EAPKGYSLEDLLATV | 60.18 | DEVSEPTHPESDEKE | 65.29 |
| TAAGIDSKLAKQESL | 61.55 | AYAKEKGLTPPSTDH | | 60.71 | PKGYSLEDLLATVKY | 62.42 | EVSEPTHPESDEKEN | 68.67 |
| AAGIDSKLAKQESLS | 61.43 | KGAEAIYNRVKAAKK | | 60.45 | GYSLEDLLATVKYYV | 64.28 | VSEPTHPESDEKENH | 66.09 |
| AGIDSKLAKQESLSH | 62.01 | AEAIYNRVKAAKKVP | | 63.69 | YSLEDLLATVKYYVE | 70.99 | SEPTHPESDEKENHV | 64.58 |
| GIDSKLAKQESLSHK | 67.50 | EAIYNRVKAAKKVPL | | 67.67 | SLEDLLATVKYYVEH | 71.99 | EPTHPESDEKENHVG | 78.08 |
| IDSKLAKQESLSHKL | 65.33 | AIYNRVKAAKKVPLD | | 64.22 | LEDLLATVKYYVEHP | 73.19 | PTHPESDEKENHVGL | 88.62 |
| DSKLAKQESLSHKLG | 70.50 | AKKVPLDRMPYNLQY | | 62.98 | EDLLATVKYYVEHPN | 78.23 | THPESDEKENHVGLN | 76.27 |
| SKLAKQESLSHKLGA | 76.16 | VPLDRMPYNLQYTVE | | 71.53 | DLLATVKYYVEHPNE | 76.15 | HPESDEKENHVGLNP | 74.54 |
| KLAKQESLSHKLGAK | 65.82 | PLDRMPYNLQYTVEV | | 72.03 | LLATVKYYVEHPNER | 66.77 | PESDEKENHVGLNPS | 77.70 |
| LAKQESLSHKLGAKK | 65.00 | LDRMPYNLQYTVEVK | | 77.77 | ATVKYYVEHPNERPH | 71.91 | ESDEKENHVGLNPSA | 76.47 |
| SHKLGAKKTDLPSSD | 64.17 | DRMPYNLQYTVEVKN | | 72.07 | TVKYYVEHPNERPHS | 73.60 | SDEKENHVGLNPSAD | 73.15 |
| LGAKKTDLPSSDREF | 65.00 | RMPYNLQYTVEVKNG | | 71.80 | VKYYVEHPNERPHSD | 74.39 | DEKENHVGLNPSADN | 60.92 |
| TDLPSSDREFYNKAY | 63.93 | MPYNLQYTVEVKNGS | | 72.71 | KYYVEHPNERPHSDN | 60.65 | PSADNLYKPSTDTEE | 61.39 |
| EFYNKAYDLLARIHQ | 64.42 | PYNLQYTVEVKNGSL | | 66.99 | VEHPNERPHSDNGFG | 65.58 | TDEAEIPQVEHSVIN | 61.07 |
| FYNKAYDLLARIHQD | 63.06 | YNLQYTVEVKNGSLI | | 63.80 | ASDHVQRNKNGQADT | 63.73 | IRQNAVETLTGLKSS | 69.63 |
| YNKAYDLLARIHQDL | 71.92 | NLQYTVEVKNGSLII | | 62.61 | SDHVQRNKNGQADTN | 80.34 | RQNAVETLTGLKSSL | 78.04 |
| NKAYDLLARIHQDLL | 62.76 | LQYTVEVKNGSLIIP | | 61.58 | DHVQRNKNGQADTNQ | 69.28 | QNAVETLTGLKSSLL | 68.19 |
| KAYDLLARIHQDLLD | 62.81 | QYTVEVKNGSLIIPH | | 64.34 | QRNKNGQADTNQTEK | 71.60 | LKSSLLLGTKDNNTI | 61.49 |
| AYDLLARIHQDLLDN | 68.17 | YTVEVKNGSLIIPHY | | 64.80 | RNKNGQADTNQTEKP | 70.27 | KSSLLLGTKDNNTIS | 60.55 |
| YDLLARIHQDLLDNK | 68.29 | TVEVKNGSLIIPHYD | | 64.16 | NKNGQADTNQTEKPN | 71.94 | SSLLLGTKDNNTISA | 63.19 |
| DLLARIHQDLLDNKG | 74.91 | VEVKNGSLIIPHYDH | | 61.67 | KNGQADTNQTEKPNE | 68.52 | SLLLGTKDNNTISAE | 61.01 |
| LLARIHQDLLDNKGR | 71.56 | EVKNGSLIIPHYDHY | | 62.31 | NGQADTNQTEKPNEE | 60.57 | LLLGTKDNNTISAEV | 66.68 |
| LARIHQDLLDNKGRQ | 78.06 | VKNGSLIIPHYDHYH | | 61.98 | EEKPQTEKPEEDKEH | 62.24 | LLGTKDNNTISAEVD | 76.60 |
| ARIHQDLLDNKGRQV | 69.86 | IIPHYDHYHNIKFEW | | 72.02 | EKPQTEKPEEDKEHD | 61.25 | LGTKDNNTISAEVDS | 78.98 |
| IHQDLLDNKGRQVDF | 60.62 | IPHYDHYHNIKFEWF | | 64.10 | KPQTEKPEEDKEHDE | 73.39 | GTKDNNTISAEVDSL | 80.97 |
| HQDLLDNKGRQVDFE | 65.15 | PHYDHYHNIKFEWFD | | 68.93 | PQTEKPEEDKEHDEV | 72.55 | TKDNNTISAEVDSLL | 73.94 |
| ALDNLLERLKDVSSD | 61.23 | HYDHYHNIKFEWFDE | | 68.13 | QTEKPEEDKEHDEVS | 74.97 | KDNNTISAEVDSLLA | 66.53 |
| LDNLLERLKDVSSDK | 73.89 | YDHYHNIKFEWFDEG | | 72.78 | TEKPEEDKEHDEVSE | 62.91 | LALLKESQPTPIQ | 62.40 |
| DNLLERLKDVSSDKV | 69.96 | DHYHNIKFEWFDEGL | | 70.32 | KPEEDKEHDEVSEPT | 68.83 | ALLKESQPTPIQ | 64.24 |
| NLLERLKDVSSDKVK | 67.03 | IKFEWFDEGLYEAPK | | 67.72 | PEEDKEHDEVSEPTH | 68.26 |  |  |
| LERLKDVSSDKVKLV | 62.39 | KFEWFDEGLYEAPKG | | 60.93 | EEDKEHDEVSEPTHP | 71.35 |  |  |
| ABCpred | | | | | | | | |
| Sequence | **Score** | **Sequence** | | **Score** | **Sequence** | **Score** | **Sequence** | **Score** |
| EKPQTEKPEEDKEHDE | 0.95 | FGNASDHVQRNKNGQA | | 0.87 | HVQRNKNGQADTNQTE | 0.80 | GQADTNQTEKPNEEKP | 0.72 |
| DPRDITSDEGDAYVTP | 0.95 | TDLPSSDREFYNKAYD | | 0.86 | PEEDKEHDEVSEPTHP | 0.79 | AYAKEKGLTPPSTDHQ | 0.71 |
| TDHQDSGNTEAKGAEA | 0.93 | HSVINAKIAEAEALLE | | 0.86 | NGVPRYIPAKDLSAET | 0.79 | LLGTKDNNTISAEVDS | 0.69 |
| EVSEPTHPESDEKENH | 0.92 | EGLYEAPKGYSLEDLL | | 0.86 | SHWIKKDSLSEAERAA | 0.79 | SSIRQNAVETLTGLKS | 0.69 |
| HYHNIKFEWFDEGLYE | 0.92 | GAEAIYNRVKAAKKVP | | 0.85 | KPNAQITYTDDEIQVA | 0.79 | SAEVDSLLALLKESQP | 0.66 |
| GLTPPSTDHQDSGNTE | 0.91 | ERAAAQAYAKEKGLTP | | 0.85 | LARIHQDLLDNKGRQV | 0.76 | ERLKDVSSDKVKLVDD | 0.66 |
| KYTTEDGYIFDPRDIT | 0.89 | TGLKSSLLLGTKDNNT | | 0.84 | EEAEDTTDEAEIPQVE | 0.76 | KAAKKVPLDRMPYNLQ | 0.65 |
| LAPIRHPERLGKPNAQ | 0.89 | SDEGDAYVTPHMTHSH | | 0.84 | YVTPHMTHSHWIKKDS | 0.75 | RMPYNLQYTVEVKNGS | 0.60 |
| VRKVGDGYVFEENGVP | 0.88 | DFEALDNLLERLKDVS | | 0.83 | GLNPSADNLYKPSTDT | 0.74 | KGYSLEDLLATVKYYV | 0.53 |
| VEHPNERPHSDNGFGN | 0.88 | PSTDTEETEEEAEDTT | | 0.83 | SDEKENHVGLNPSADN | 0.74 |  |  |
| GSLIIPHYDHYHNIKF | 0.88 | KLAKQESLSHKLGAKK | | 0.81 | LSAETAAGIDSKLAKQ | 0.74 |  |  |
| DDEIQVAKLAGKYTTE | 0.88 | AEALLEKVTDSSIRQN | | 0.81 | EIPQVEHSVINAKIAE | 0.72 |  |  |
| Emini surface accessibility Prediction | | | | | | | | |
| Sequence Sequence Sequence Sequence | | | | | | | | |
| SSDREFYNKA | | PSTDHQDSG | | | HPNERPHS | | HVQRNKNG | |
| DTNQTEKPNEEKPQTEKPEEDKEH | | PTHPESDEKE | | | NLYKPSTDTEETEEEAEDTTD | |  | |
|  |  |  | |  |  |  |  |  |
| Ellipro | | | | | | | | |
| Sequence | **Score** | | **Sequence** | **Score** | **Sequence** | **Score** | **Sequence** | **Score** |
| LEKVTDSSIRQNAVETLTGLKSSLLLGTKDNNTISAEVDSLLALLKESQPTPIQ | 0.826 | | EAKGAEAIYNRVKAAK | 0.638 | SDHVQRNKNGQADTNQTEK | 0.605 | EPTH | 0.555 |
| EKLVKEAVRKVGDGYVFEENGVPRYIPAKDLSAETAAGIDSKLAKQESL | 0.801 | | EHPNERPHSDNGF | 0.611 | DLLDNKGRQVDFEALDNLLERLKDVSSDKVKLVDDILAFLAPIR | 0.589 | LIIPHYDH | 0.546 |
| TEETEEEAEDTTDEAEIPQVEH | 0.79 | | KTDLPSSDREFYN | 0.608 | AKEKGL | 0.558 | DEGLY | 0.527 |
| *LBtope assigns scores between 0-100% to each of the identified epitopes. In this study, only those epitopes with the scores above 60% are considered. | | | | | | | | |

**Supplementary Table S10. The predicted discontinuous B cell epitopes of PhtD-C.**

| Ellipro |  |  |
| --- | --- | --- |
| Residues | **Number of residues** | **Score** |
| E1, K2, L3, V4, K5, E6 | 6 | 0.975 |
| E298, H299, P300, N301, E302, R303, P304, H305, S306, D307, N308, G309, F310, N334, E354, P355, T356, H357, P358, H366, V367, G368, L369, T383, E384, E385, T386, E387, E388, E389, A390, E391, D392, T393, T394, D395, E396, A397, E398, I399, P400, Q401, V402, E403, H404, S405, N408, L418, E419, K420, V421, T422, D423, S424, S425, I426, R427, Q428, N429, A430, V431, E432, T433, L434, T435, G436, L437, K438, S439, S440, L441, L442, L443, G444, T445, K446, D447, N448, N449, T450, I451, S452, A453, E454, V455, D456, S457, L458, L459, A460, L461, L462, K463, E464, S465, Q466, P467, T468, P469, I470, Q471 | 101 | 0.746 |
| A7, V8, K10, V11, G12, D13, G14, Y15, V16, F17, E18, E19, N20, G21, V22, P23, R24, Y25, I26, P27, A28, K29, D30, L31, S32, A33, E34, T35, A36, A37, G38, I39, D40, S41, K42, L43, A44, K45, Q46, E47, S48, L49, A55, K56, K57, T58, D59, L60, P61, S62, S63, D64, R65, E66, F67, Y68, N69, K70, K103, D104, V105, S106, S107, D108, K109, K111, L112, V113, D114, D115, I116, A118, F119, L120, A121, P122, I123, H125, E127, Q144, K147 | 81 | 0.667 |
| Y158, P162, R163, I165, T166, K204, G205, L206, T207, G217, N218, E220, A221, K222, G223, A224, E225, A226, I227, Y228, N229, R230, V231, K232, A233, A234, K235, D274, E275, G276, L277, Y278, N322, G323, Q324, A325, D326, T327, N328, Q329, T330, E331, K332 | 43 | 0.608 |
| D81, L82, L83, D84, N85, K86, G87, R88, Q89, V90, D91, F92, E93, A94, L95, D96, N97, L98, L99, R101, E169, A172, Y173, V174, T175, P176, H177, H182, Y200, A201, K202, E203 | 32 | 0.584 |
| S256, L257, I258, I259, P260, H261, Y262, D263, H264, S314, D315, H316, V317, N320, K321 | 15 | 0.526 |
| Discotope |  |  |
| Residues | **Number of residues** | **Score** |
| T175, H177, M178, T179, H180, S181, H182, W183, K185, K186, D187, S188, L189, S190, E191, A192, E193, R194, A195, A196, A197, Q198, A199, Y200, A201, K202, E203, K204, G205, L206, T207, P208, P209, S210, T211, D212, H213, Q214, D215, S216, G217, N218, T219, E220, A221, K222, G223, A224, E225, A226, I227, Y228, N229, R230, V231, K232, A233, A234, K235, K236, V237, P238, L239, D240, R241, M242, P243, Y244, N245, L246, Q247, Y248, T249, V250, E251, V252, K253, N254, G255, S256, L257, I258, I259, P260, H261, Y262, D263, H264, Y265, H266, N267, I268, K269, F270, E271, W272, F273, D274, E275, G276, L277, Y278, E279, A280, P281, K282, G283, Y284, S285, L286, E287, D288, L289, L290, A291, T292, V293, K294, Y295, Y296, V297, E298, H299, P300, N301, E302, R303, P304, H305, S306, D307, N308, G309, F310, G311, N312, A313, S314, D315, H316, V317, Q318, R319, N320, K321, N322, G323, Q324, A325, D326, T327, N328, Q329, T330, E331, K332, P333, N334, E335, E336, K337, P338, Q339, T340, E341, K342, P343, E344, E345, D346, K347, E348, H349, D350, E351, V352, S353, E354, P355, T356, H357, P358, E359, S360, D361, E362, K363, E6364, N365 | 189 | 3.71 |
| N370, S372, A373, D374, N375, L376, Y377, K378, P379 | 9 | -0.85 |
| T386, E387, E388, E389, A390, E391, D392, T393, T394, D395, E396, E398 | 12 | -0.89 |
| E19, G21, V22, P23, R24, Y25 | 6 | -1.01 |
| K45, E47, S48, L49, S50 | 5 | -1.49 |
| K86, G87, R88, Q89 | 4 | -1.53 |
| F67, N69, K70, A71 | 4 | -1.80 |
| D141, I143, V145, A146, L148, G150, Y152, T154, E155, D156, G157, Y158, I159, F160, D161, P162, R163, D164, I165, T166, D168, E169 | 22 | -1.86 |
| I123, R124, H125, E127, R128, L129 | 6 | -1.98 |
| E1, K2, L3, V4, K5, E6, A7, V8 | 8 | -2.22 |
| D447, N448, N449, T450, I451, S452, A453, E454 | 8 | -2.33 |

**Supplementary Table S11.** **Prediction of MHC-II epitopes of PspA2-A and PspA1-5-B.** The peptides with IEDB percentile rank <10.0 and NetMHCIIpan rank value <1.0 were considered for the next analysis.

| PspA2-A | IEDB | | |  | | |
| --- | --- | --- | --- | --- | --- | --- |
|  | **Allele** | **Peptide** | **Percentile Rank** | **Allele** | **Peptide** | **Percentile Rank** |
|  | H2-IEd | QQAYLAYQRASNKAE | 4.30 | DRB1*08:01 | AYLAYQRASNKAEAA | 0.74 |
|  | H2-IEd | VQQAYLAYQRASNKA | 4.60 | DRB1*08:01 | EEVQQAYLAYQRASN | 0.74 |
|  | H2-IEd | QAYLAYQRASNKAEA | 4.85 | DRB1*08:01 | EVQQAYLAYQRASNK | 0.74 |
|  | H2-IEd | EVQQAYLAYQRASNK | 5.05 | DRB1*08:01 | QAYLAYQRASNKAEA | 0.74 |
|  | H2-IEd | AYLAYQRASNKAEAA | 5.80 | DRB1*08:01 | QQAYLAYQRASNKAE | 0.74 |
|  | H2-IAd | TEEVQQAYLAYQRAS | 7.30 | DRB1*08:01 | VQQAYLAYQRASNKA | 0.74 |
|  | H2-IAb | AYLAYQRASNKAEAA | 7.45 | DRB1*08:01 | YLAYQRASNKAEAAK | 0.74 |
|  | H2-IAd | EEVQQAYLAYQRASN | 7.45 | DRB1*04:01 | QAYLAYQRASNKAEA | 4.10 |
|  | H2-IAd | ATEEVQQAYLAYQRA | 7.90 | DRB1*04:01 | AYLAYQRASNKAEAA | 6.10 |
|  | H2-IAb | YLAYQRASNKAEAAK | 8.20 | DRB1*04:01 | QQAYLAYQRASNKAE | 6.20 |
|  | H2-IEd | EEVQQAYLAYQRASN | 8.40 | DRB1*11:01 | QQAYLAYQRASNKAE | 8.40 |
|  | H2-IAd | EVQQAYLAYQRASNK | 8.90 | DRB1*11:01 | QAYLAYQRASNKAEA | 8.70 |
|  | H2-IAd | VQQAYLAYQRASNKA | 9.10 | DRB1*04:01 | YLAYQRASNKAEAAK | 9.60 |
|  | **NetMHCIIpan** | | |  | | |
|  | **Allele** | **Peptide** | **%Rank** | **Allele** | **Peptide** | **%Rank** |
|  | H-2-IEd | DAAVKKSEAAKKAYE | 0.71 | DRB1_0801 | KDYDAAVKKSEAAKK | 0.39 |
|  | H-2-IEd | AKKAYEEAKKALEEA | 0.70 | DRB1_0801 | EAAKKAYEEAKKALE | 0.46 |
|  | H-2-IEd | KKAYEEAKKALEEAK | 0.20 | DRB1_0801 | AAKKAYEEAKKALEE | 0.03 |
|  | H-2-IEd | AKKALEEAKVAQKKY | 0.87 | DRB1_0801 | AKKAYEEAKKALEEA | 0.01 |
|  | H-2-IEd | KKALEEAKVAQKKYE | 0.45 | DRB1_0801 | KKAYEEAKKALEEAK | 0.00 |
|  | H-2-IEd | VQQAYLAYQRASNKA | 0.63 | DRB1_0801 | KAYEEAKKALEEAKV | 0.05 |
|  | H-2-IEd | QQAYLAYQRASNKAE | 0.25 | DRB1_0801 | QQAYLAYQRASNKAE | 0.75 |
|  | H-2-IEd | QAYLAYQRASNKAEA | 0.69 | DRB1_1101 | EKDYDAAVKKSEAAK | 0.39 |
|  | H-2-IAd | LEKEASEAIAKATEE | 0.70 | DRB1_1101 | AAKKAYEEAKKALEE | 0.48 |
|  | H-2-IAd | EKEASEAIAKATEEV | 0.93 | DRB1_1101 | AKKAYEEAKKALEEA | 0.18 |
|  | DRB1_0801 | KAEKDYDAAVKKSEA | 0.25 | DRB1_1101 | KKAYEEAKKALEEAK | 0.07 |
|  | DRB1_0801 | AEKDYDAAVKKSEAA | 0.10 | DRB1_1101 | KAYEEAKKALEEAKV | 0.42 |
|  | DRB1_0801 | EKDYDAAVKKSEAAK | 0.06 | DRB1_1101 | QQAYLAYQRASNKAE | 0.96 |
| PspA1-B | **IEDB** | | |  | | |
|  | **Allele** | **Peptide** | **Percentile Rank** | **Allele** | **Peptide** | **Percentile Rank** |
|  | H2-IAb | YVKEGFRAPLQSELD | 8.60 | H2-IAb | DYVKEGFRAPLQSEL | 8.90 |
|  | **NetMHCIIpan** | | |  | | |
|  | **Allele** | **Peptide** | **%Rank** | **Allele** | **Peptide** | **%Rank** |
|  | H-2-IAb | KEGFRAPLQSELDAK | 0.95 | DRB1_0101 | SDKIDELDAEIAKLE | 0.59 |
|  | H-2-IAd | YLAAAEEDLIAKKAE | 0.61 | DRB1_0401 | VEDFKNSDGEQAGQY | 0.98 |
|  | H-2-IAd | LAAAEEDLIAKKAEL | 0.59 | DRB1_0801 | AAEEDLIAKKAELEQ | 0.95 |
|  | H-2-IAd | AAAEEDLIAKKAELE | 0.31 | DRB1_0801 | AEEDLIAKKAELEQT | 0.66 |
|  | DRB1_0101 | LSDKIDELDAEIAKL | 0.95 | DRB1_0801 | EEDLIAKKAELEQTE | 0.50 |
| PspA2-B | **IEDB** | | |  | | |
|  | **Allele** | **Peptide** | **Percentile Rank** | **Allele** | **Peptide** | **Percentile Rank** |
|  | H2-IAb | YAKEGFRAPLQSKLD | 6.50 | H2-IAb | AKEGFRAPLQSKLDA | 6.95 |
|  | H2-IAb | DYAKEGFRAPLQSKL | 6.60 | H2-IAb | KEGFRAPLQSKLDAK | 7.90 |
|  | **NetMHCIIpan** | | |  | | |
|  | **Allele** | **Peptide** | **%Rank** | **Allele** | **Peptide** | **%Rank** |
|  | H-2-IEd | EKTIAAKKTELEKTE | 0.67 | DRB1_0801 | GLEKTIAAKKTELEK | 0.38 |
|  | H-2-IAd | STEGLEKTIAAKKTE | 0.49 | DRB1_0801 | LEKTIAAKKTELEKT | 0.25 |
|  | H-2-IAd | TEGLEKTIAAKKTEL | 0.42 | DRB1_0801 | EKTIAAKKTELEKTE | 0.19 |
|  | H-2-IAd | EGLEKTIAAKKTELE | 0.20 | DRB1_0801 | KTIAAKKTELEKTEA | 0.61 |
|  | DRB1_0101 | LSDKIDELDAEIAKL | 0.95 | DRB1_1501 | ENNNVEDYSTEGLEK | 0.65 |
|  | DRB1_0101 | SDKIDELDAEIAKLE | 0.59 | DRB1_1501 | NNNVEDYSTEGLEKT | 0.51 |
| PspA3-B | **NetMHCIIpan** | | |  | | |
|  | **Allele** | **Peptide** | **%Rank** | **Allele** | **Peptide** | **%Rank** |
|  | DRB1_0801 | TKKAEFEKTQKELDA | 0.15 | DRB1_0801 | KAEFEKTQKELDAAL | 0.08 |
|  | DRB1_0801 | KKAEFEKTQKELDAA | 0.06 | DRB1_0801 | AEFEKTQKELDAALN | 0.59 |
| PspA4-B | **NetMHCIIpan** | | |  | | |
|  | **Allele** | **Peptide** | **%Rank** | **Allele** | **Peptide** | **%Rank** |
|  | H-2-IEd | EEAIATKKAELEKTQ | 0.75 | DRB1_0101 | NEKVEALQNQVAELE | 0.93 |
|  | H-2-IAd | YIKEGLEEAIATKKA | 0.89 | DRB1_0401 | NEKVEALQNQVAELE | 0.96 |
|  | H-2-IAd | IKEGLEEAIATKKAE | 0.06 | DRB1_0801 | GLEEAIATKKAELEK | 0.97 |
|  | H-2-IAd | KEGLEEAIATKKAEL | 0.05 | DRB1_0801 | LEEAIATKKAELEKT | 0.68 |
|  | H-2-IAd | EGLEEAIATKKAELE | 0.02 | DRB1_0801 | EEAIATKKAELEKTQ | 0.56 |
|  | H-2-IAd | GLEEAIATKKAELEK | 0.19 | DRB1_0801 | KKAELEKTQKELDAA | 0.73 |
| PspA5-B | **IEDB** | | |  | | |
|  | **Allele** | **Peptide** | **Percentile Rank** | **Allele** | **Peptide** | **Percentile Rank** |
|  | DRB1*03:01 | VTRLQSDLKDAEENN | 3.30 | DRB1*03:01 | DKEVTRLQSDLKDAE | 5.70 |
|  | DRB1*03:01 | EVTRLQSDLKDAEEN | 3.40 | DRB1*03:01 | RLQSDLKDAEENNVE | 6.90 |
|  | DRB1*03:01 | KEVTRLQSDLKDAEE | 4.10 | DRB1*03:01 | LQSDLKDAEENNVED | 7.20 |
|  | DRB1*03:01 | TRLQSDLKDAEENNV | 4.50 |  |  |  |
|  | **NetMHCIIpan** | | |  | | |
|  | **Allele** | **Peptide** | **%Rank** | **Allele** | **Peptide** | **%Rank** |
|  | H-2-IAd | AAEDANIEALQNKVA | 0.72 | DRB1_0801 | KVELNNTQKALDTAP | 0.43 |
|  | H-2-IAd | AEDANIEALQNKVAD | 0.99 | DRB1_0801 | TQKALDTAPKALDTA | 0.61 |
|  | DRB1_0101 | DANIEALQNKVADLE | 0.72 | DRB1_0801 | QKALDTAPKALDTAL | 0.83 |
|  | DRB1_0301 | EVTRLQSDLKDAEEN | 0.75 | DRB1_1501 | AEDANIEALQNKVAD | 0.96 |
|  | DRB1_0301 | VTRLQSDLKDAEENN | 0.53 | DRB1_1501 | EDANIEALQNKVADL | 0.80 |
|  | DRB1_0801 | KKVELNNTQKALDTA | 0.79 | DRB1_1501 | DANIEALQNKVADLE | 0.62 |

**Supplementary Table S12.** **Prediction of MHC-II epitopes of PhtD-C.** The epitopes with IEDB percentile rank <10.0 and NetMHCIIpan rank value <1.0 were considered for the further analysis.

| IEDB | | |  | | | | |
| --- | --- | --- | --- | --- | --- | --- | --- |
| Allele | **Peptide** | **Percentile Rank** | **Allele** | **Peptide** | | **Percentile Rank** | |
| H2-IEd | AEAIYNRVKAAKKVP | 2.40 | DRB1*11:01 | AEAIYNRVKAAKKVP | | 4.50 | |
| H2-IEd | GAEAIYNRVKAAKKV | 3.00 | DRB1*11:01 | EAIYNRVKAAKKVPL | | 4.60 | |
| H2-IEd | KGAEAIYNRVKAAKK | 3.25 | DRB1*11:01 | GAEAIYNRVKAAKKV | | 4.60 | |
| H2-IEd | EAIYNRVKAAKKVPL | 3.50 | DRB1*03:01 | RDITSDEGDAYVTPH | | 4.60 | |
| H2-IAd | QNAVETLTGLKSSLL | 4.30 | DRB1*04:01 | DSKLAKQESLSHKLG | | 4.70 | |
| H2-IAd | NAVETLTGLKSSLLL | 4.40 | DRB1*04:01 | SKLAKQESLSHKLGA | | 4.70 | |
| H2-IEd | MTHSHWIKKDSLSEA | 4.85 | DRB1*04:01 | IDSKLAKQESLSHKL | | 4.80 | |
| H2-IAb | DEGDAYVTPHMTHSH | 5.00 | DRB1*11:01 | AIYNRVKAAKKVPLD | | 4.90 | |
| H2-IEd | HMTHSHWIKKDSLSE | 5.00 | DRB1*04:01 | GIDSKLAKQESLSHK | | 5.50 | |
| H2-IEd | ILAFLAPIRHPERLG | 5.10 | DRB1*03:01 | SDKVKLVDDILAFLA | | 5.50 | |
| H2-IAb | EGDAYVTPHMTHSHW | 5.20 | DRB1*07:01 | DSLLALLKESQPTPI | | 5.60 | |
| H2-IEd | AIYNRVKAAKKVPLD | 5.80 | DRB1*13:01 | EKLVKEAVRKVGDGY | | 5.70 | |
| H2-IAd | AVETLTGLKSSLLLG | 6.05 | DRB1*13:01 | KLVKEAVRKVGDGYV | | 5.70 | |
| H2-IEd | IYNRVKAAKKVPLDR | 6.05 | DRB1*13:01 | LVKEAVRKVGDGYVF | | 5.70 | |
| H2-IAb | SDEGDAYVTPHMTHS | 6.20 | DRB1*03:01 | DKVKLVDDILAFLAP | | 5.90 | |
| H2-IAd | LSEAERAAAQAYAKE | 6.25 | DRB1*03:01 | KVKLVDDILAFLAPI | | 6.20 | |
| H2-IEd | AKGAEAIYNRVKAAK | 6.30 | DRB1*04:01 | GDGYVFEENGVPRYI | | 6.30 | |
| H2-IAb | GDAYVTPHMTHSHWI | 6.40 | DRB1*11:01 | IYNRVKAAKKVPLDR | | 6.50 | |
| H2-IAd | SEAERAAAQAYAKEK | 6.50 | DRB1*01:01 | LTGLKSSLLLGTKDN | | 6.80 | |
| H2-IAb | VDDILAFLAPIRHPE | 6.50 | DRB1*11:01 | KGAEAIYNRVKAAKK | | 6.90 | |
| H2-IAb | DDILAFLAPIRHPER | 6.65 | DRB1*03:01 | SSDKVKLVDDILAFL | | 6.90 | |
| H2-IEd | YNRVKAAKKVPLDRM | 6.70 | DRB1*15:01 | VKLVDDILAFLAPIR | | 7.20 | |
| H2-IAd | SLSEAERAAAQAYAK | 6.85 | DRB1*07:01 | AIYNRVKAAKKVPLD | | 7.40 | |
| H2-IEd | LAFLAPIRHPERLGK | 7.20 | DRB1*04:01 | DGYVFEENGVPRYIP | | 7.40 | |
| H2-IAb | DAYVTPHMTHSHWIK | 7.70 | DRB1*07:01 | EAIYNRVKAAKKVPL | | 7.40 | |
| H2-IAb | DILAFLAPIRHPERL | 7.80 | DRB1*08:01 | AEAIYNRVKAAKKVP | | 7.60 | |
| H2-IEd | THSHWIKKDSLSEAE | 8.10 | DRB1*08:01 | AIYNRVKAAKKVPLD | | 7.60 | |
| H2-IAd | AIYNRVKAAKKVPLD | 8.15 | DRB1*08:01 | AKGAEAIYNRVKAAK | | 7.60 | |
| H2-IEd | NRVKAAKKVPLDRMP | 8.15 | DRB1*08:01 | EAIYNRVKAAKKVPL | | 7.60 | |
| H2-IAb | GAEAIYNRVKAAKKV | 8.20 | DRB1*08:01 | GAEAIYNRVKAAKKV | | 7.60 | |
| H2-IAb | AEAIYNRVKAAKKVP | 8.25 | DRB1*08:01 | IYNRVKAAKKVPLDR | | 7.60 | |
| H2-IEd | DILAFLAPIRHPERL | 8.35 | DRB1*08:01 | KGAEAIYNRVKAAKK | | 7.60 | |
| H2-IAd | EAERAAAQAYAKEKG | 8.35 | DRB1*11:01 | DDILAFLAPIRHPER | | 8.00 | |
| H2-IAd | VETLTGLKSSLLLGT | 8.35 | DRB1*11:01 | DEIQVAKLAGKYTTE | | 8.00 | |
| H2-IEd | AFLAPIRHPERLGKP | 8.65 | DRB1*11:01 | DILAFLAPIRHPERL | | 8.00 | |
| H2-IEd | PHMTHSHWIKKDSLS | 8.70 | DRB1*11:01 | ILAFLAPIRHPERLG | | 8.00 | |
| H2-IAb | GNTEAKGAEAIYNRV | 8.90 | DRB1*11:01 | LAFLAPIRHPERLGK | | 8.00 | |
| H2-IAb | SGNTEAKGAEAIYNR | 8.90 | DRB1*03:01 | VKLVDDILAFLAPIR | | 8.00 | |
| H2-IAb | DSGNTEAKGAEAIYN | 8.95 | DRB1*08:01 | EENGVPRYIPAKDLS | | 8.10 | |
| H2-IAd | EAIYNRVKAAKKVPL | 9.10 | DRB1*08:01 | ENGVPRYIPAKDLSA | | 8.10 | |
| H2-IAb | KGAEAIYNRVKAAKK | 9.10 | DRB1*08:01 | FEENGVPRYIPAKDL | | 8.10 | |
| H2-IEd | RVKAAKKVPLDRMPY | 9.50 | DRB1*08:01 | GVPRYIPAKDLSAET | | 8.10 | |
| H2-IEd | DDILAFLAPIRHPER | 9.80 | DRB1*08:01 | NGVPRYIPAKDLSAE | | 8.10 | |
| H2-IAb | SLLALLKESQPTPIQ | 9.85 | DRB1*03:01 | RLKDVSSDKVKLVDD | | 8.10 | |
| H2-IAb | LVDDILAFLAPIRHP | 9.95 | DRB1*08:01 | VFEENGVPRYIPAKD | | 8.10 | |
| DRB1*01:01 | AVETLTGLKSSLLLG | 0.44 | DRB1*08:01 | VPRYIPAKDLSAETA | | 8.10 | |
| DRB1*01:01 | NAVETLTGLKSSLLL | 0.51 | DRB1*15:01 | ETLTGLKSSLLLGTK | | 8.20 | |
| DRB1*01:01 | VETLTGLKSSLLLGT | 0.52 | DRB1*07:01 | GAEAIYNRVKAAKKV | | 8.20 | |
| DRB1*01:01 | ETLTGLKSSLLLGTK | 0.96 | DRB1*07:01 | IYNRVKAAKKVPLDR | | 8.20 | |
| DRB1*01:01 | QNAVETLTGLKSSLL | 1.60 | DRB1*07:01 | YNRVKAAKKVPLDRM | | 8.20 | |
| DRB1*08:01 | HMTHSHWIKKDSLSE | 1.70 | DRB1*04:01 | VGDGYVFEENGVPRY | | 8.30 | |
| DRB1*08:01 | HSHWIKKDSLSEAER | 1.70 | DRB1*01:01 | AEAIYNRVKAAKKVP | | 8.40 | |
| DRB1*08:01 | HWIKKDSLSEAERAA | 1.70 | DRB1*01:01 | AIYNRVKAAKKVPLD | | 8.40 | |
| DRB1*08:01 | MTHSHWIKKDSLSEA | 1.70 | DRB1*01:01 | EAIYNRVKAAKKVPL | | 8.40 | |
| DRB1*08:01 | SHWIKKDSLSEAERA | 1.70 | DRB1*01:01 | GAEAIYNRVKAAKKV | | 8.40 | |
| DRB1*08:01 | THSHWIKKDSLSEAE | 1.70 | DRB1*01:01 | KGAEAIYNRVKAAKK | | 8.40 | |
| DRB1*08:01 | WIKKDSLSEAERAAA | 1.70 | DRB1*15:01 | VETLTGLKSSLLLGT | | 8.60 | |
| DRB1*01:01 | TLTGLKSSLLLGTKD | 2.10 | DRB1*15:01 | ILAFLAPIRHPERLG | | 8.70 | |
| DRB1*11:01 | YNKAYDLLARIHQDL | 2.50 | DRB1*03:01 | ERLKDVSSDKVKLVD | | 8.90 | |
| DRB1*11:01 | FYNKAYDLLARIHQD | 2.60 | DRB1*15:01 | EDLLATVKYYVEHPN | | 9.00 | |
| DRB1*11:01 | NKAYDLLARIHQDLL | 2.70 | DRB1*03:01 | NTISAEVDSLLALLK | | 9.10 | |
| DRB1*11:01 | KAYDLLARIHQDLLD | 2.90 | DRB1*11:01 | YNRVKAAKKVPLDRM | | 9.10 | |
| DRB1*03:01 | DPRDITSDEGDAYVT | 3.00 | DRB1*07:01 | AEAIYNRVKAAKKVP | | 9.20 | |
| DRB1*11:01 | EFYNKAYDLLARIHQ | 3.20 | DRB1*11:01 | EKLVKEAVRKVGDGY | | 9.20 | |
| DRB1*15:01 | VDDILAFLAPIRHPE | 3.30 | DRB1*03:01 | NNTISAEVDSLLALL | | 9.20 | |
| DRB1*15:01 | DDILAFLAPIRHPER | 3.40 | DRB1*11:01 | VDDILAFLAPIRHPE | | 9.30 | |
| DRB1*03:01 | PRDITSDEGDAYVTP | 3.40 | DRB1*04:01 | GYVFEENGVPRYIPA | | 9.40 | |
| DRB1*11:01 | AYDLLARIHQDLLDN | 3.50 | DRB1*03:01 | LKDVSSDKVKLVDDI | | 9.40 | |
| DRB1*11:01 | YDLLARIHQDLLDNK | 3.50 | DRB1*15:01 | AVETLTGLKSSLLLG | | 9.70 | |
| DRB1*15:01 | LVDDILAFLAPIRHP | 3.60 | DRB1*03:01 | DNNTISAEVDSLLAL | | 9.70 | |
| DRB1*03:01 | FDPRDITSDEGDAYV | 4.10 | DRB1*03:01 | SHWIKKDSLSEAERA | | 9.70 | |
| DRB1*15:01 | KLVDDILAFLAPIRH | 4.10 | DRB1*15:01 | EAIYNRVKAAKKVPL | | 9.90 | |
| DRB1*15:01 | DILAFLAPIRHPERL | 4.30 | DRB1*03:01 | HSHWIKKDSLSEAER | | 9.90 | |
| DRB1*07:01 | SLLALLKESQPTPIQ | 4.30 |  |  | |  | |
| NetMHCIIpan |  |  |  |  |  | |  |
| Allele | **Peptide** | **%Rank** | **Allele** | **Peptide** | **%Rank** | |  |
| H-2-IEd | VPRYIPAKDLSAETA | 0.66 | DRB1_0401 | VGDGYVFEENGVPRY | 0.67 | |  |
| H-2-IEd | LATVKYYVEHPNERP | 0.21 | DRB1_0401 | GDGYVFEENGVPRYI | 0.80 | |  |
| H-2-IEd | ATVKYYVEHPNERPH | 0.05 | DRB1_0401 | NGVPRYIPAKDLSAE | 0.10 | |  |
| H-2-IEd | TVKYYVEHPNERPHS | 0.03 | DRB1_0401 | GVPRYIPAKDLSAET | 0.04 | |  |
| H-2-IEd | VKYYVEHPNERPHSD | 0.22 | DRB1_0401 | VPRYIPAKDLSAETA | 0.03 | |  |
| H-2-IAd | PRYIPAKDLSAETAA | 0.71 | DRB1_0401 | PRYIPAKDLSAETAA | 0.13 | |  |
| H-2-IAd | RYIPAKDLSAETAAG | 0.31 | DRB1_0401 | REFYNKAYDLLARIH | 0.68 | |  |
| H-2-IAd | YIPAKDLSAETAAGI | 0.37 | DRB1_0401 | GRQVDFEALDNLLER | 0.73 | |  |
| H-2-IAd | IPAKDLSAETAAGID | 0.81 | DRB1_0401 | RQVDFEALDNLLERL | 0.65 | |  |
| H-2-IAd | SAETAAGIDSKLAKQ | 0.47 | DRB1_0401 | QVDFEALDNLLERLK | 0.31 | |  |
| H-2-IAd | AETAAGIDSKLAKQE | 0.24 | DRB1_0401 | NAQITYTDDEIQVAK | 0.99 | |  |
| H-2-IAd | LSEAERAAAQAYAKE | 0.98 | DRB1_0401 | DRMPYNLQYTVEVKN | 0.90 | |  |
| H-2-IAd | SEAERAAAQAYAKEK | 0.69 | DRB1_0401 | RMPYNLQYTVEVKNG | 0.52 | |  |
| H-2-IAd | GNTEAKGAEAIYNRV | 0.77 | DRB1_0401 | DNGFGNASDHVQRNK | 0.70 | |  |
| H-2-IAd | NTEAKGAEAIYNRVK | 0.35 | DRB1_0801 | FYNKAYDLLARIHQD | 0.74 | |  |
| H-2-IAd | IPQVEHSVINAKIAE | 0.73 | DRB1_0801 | YNKAYDLLARIHQDL | 0.99 | |  |
| H-2-IAd | PQVEHSVINAKIAEA | 0.36 | DRB1_0801 | QVDFEALDNLLERLK | 0.77 | |  |
| H-2-IAd | QVEHSVINAKIAEAE | 0.23 | DRB1_0801 | HSHWIKKDSLSEAER | 0.72 | |  |
| H-2-IAd | VEHSVINAKIAEAEA | 0.35 | DRB1_1101 | EKLVKEAVRKVGDGY | 0.68 | |  |
| H-2-IAd | EHSVINAKIAEAEAL | 0.75 | DRB1_1101 | VPRYIPAKDLSAETA | 0.97 | |  |
| H-2-IAd | SIRQNAVETLTGLKS | 0.76 | DRB1_1101 | FYNKAYDLLARIHQD | 0.05 | |  |
| DRB1_0101 | PAKDLSAETAAGIDS | 0.78 | DRB1_1101 | YNKAYDLLARIHQDL | 0.06 | |  |
| DRB1_0101 | AKDLSAETAAGIDSK | 0.32 | DRB1_1101 | NKAYDLLARIHQDLL | 0.13 | |  |
| DRB1_0101 | DRMPYNLQYTVEVKN | 0.33 | DRB1_1101 | KAYDLLARIHQDLLD | 0.83 | |  |
| DRB1_0101 | RMPYNLQYTVEVKNG | 0.13 | DRB1_1101 | PYNLQYTVEVKNGSL | 0.65 | |  |
| DRB1_0301 | TEDGYIFDPRDITSD | 0.89 | DRB1_1101 | YNLQYTVEVKNGSLI | 0.46 | |  |
| DRB1_0301 | EDGYIFDPRDITSDE | 0.72 | DRB1_1101 | NLQYTVEVKNGSLII | 0.55 | |  |
| DRB1_0301 | FDPRDITSDEGDAYV | 0.70 | DRB1_1101 | VDSLLALLKESQPTP | 0.90 | |  |
| DRB1_0301 | DPRDITSDEGDAYVT | 0.41 | DRB1_1301 | KGAEAIYNRVKAAKK | 0.70 | |  |
| DRB1_0301 | PRDITSDEGDAYVTP | 0.30 | DRB1_1301 | GAEAIYNRVKAAKKV | 0.42 | |  |
| DRB1_0301 | RDITSDEGDAYVTPH | 0.85 | DRB1_1301 | AEAIYNRVKAAKKVP | 0.21 | |  |

**Supplementary Figure S1. Domain structure of PspA protein.** Major domains of PspA are α-helical charged domain (amino acids 1-288) consisting of A, Aˊ and B regions, proline-rich domain (amino acids 289-370, C region), and choline-binding domain (amino acids 371-571). Within the α-HD, region B is a clade-defining region of the PspA molecule, which is represented by the stippled box.


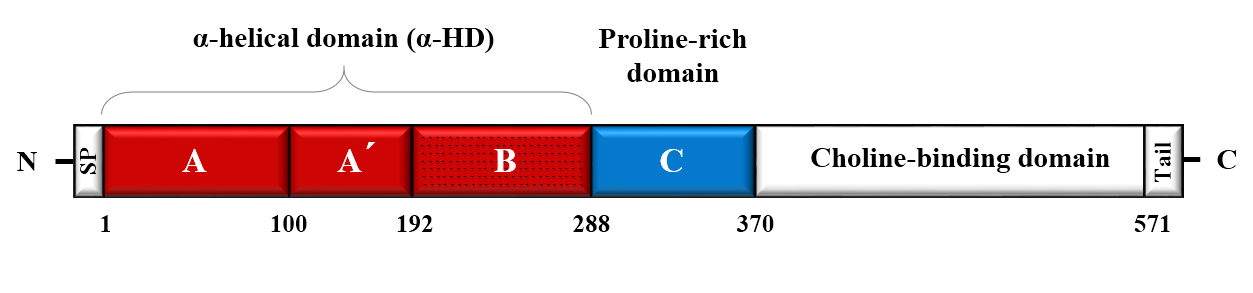

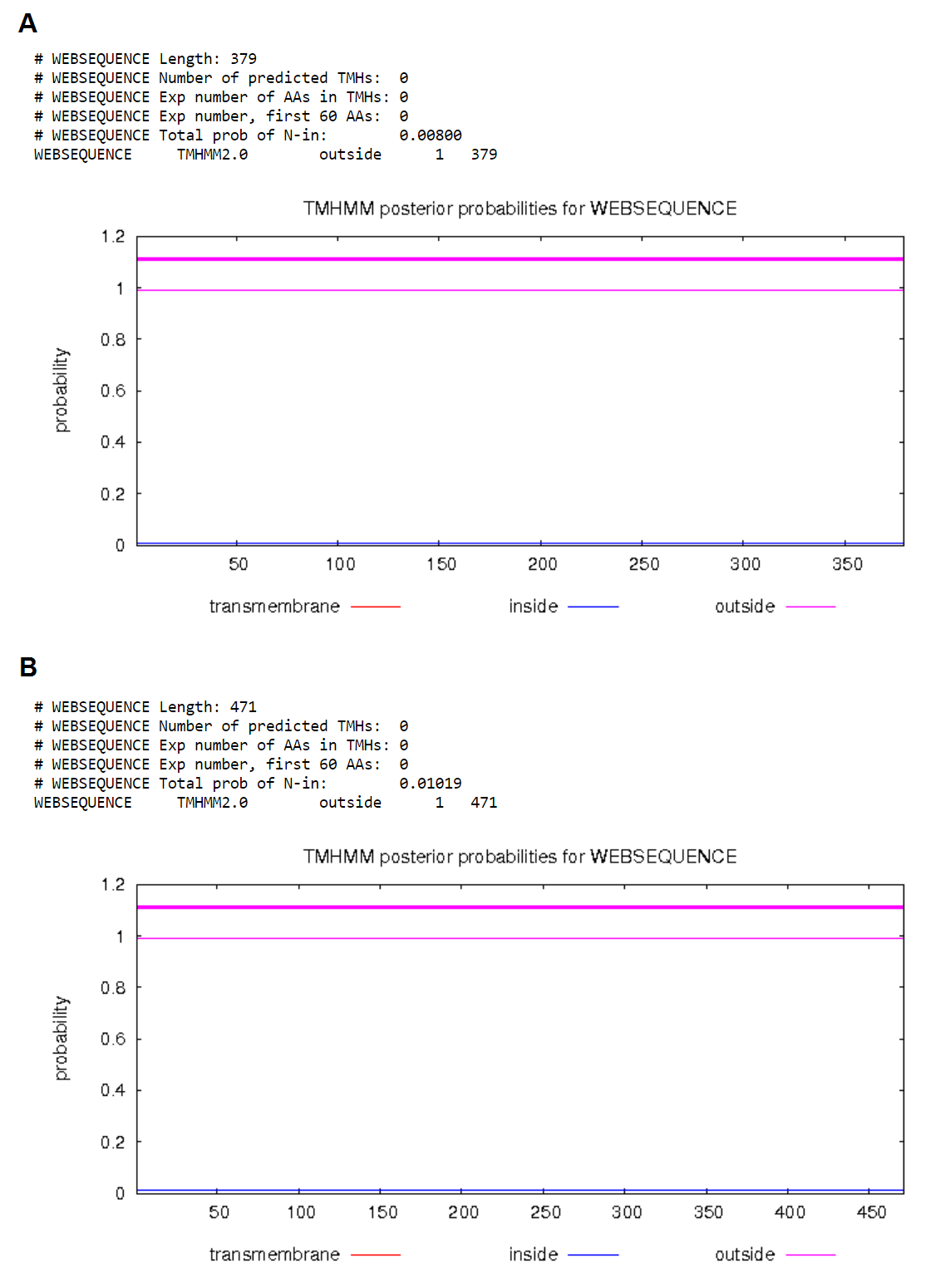


**Supplementary Figure S2. The results of transmembrane helices prediction.** A and B show the prediction results of transmembrane helices in PspA2 and PhtD-C, respectively. The pink lines indicate the domains facing outside.


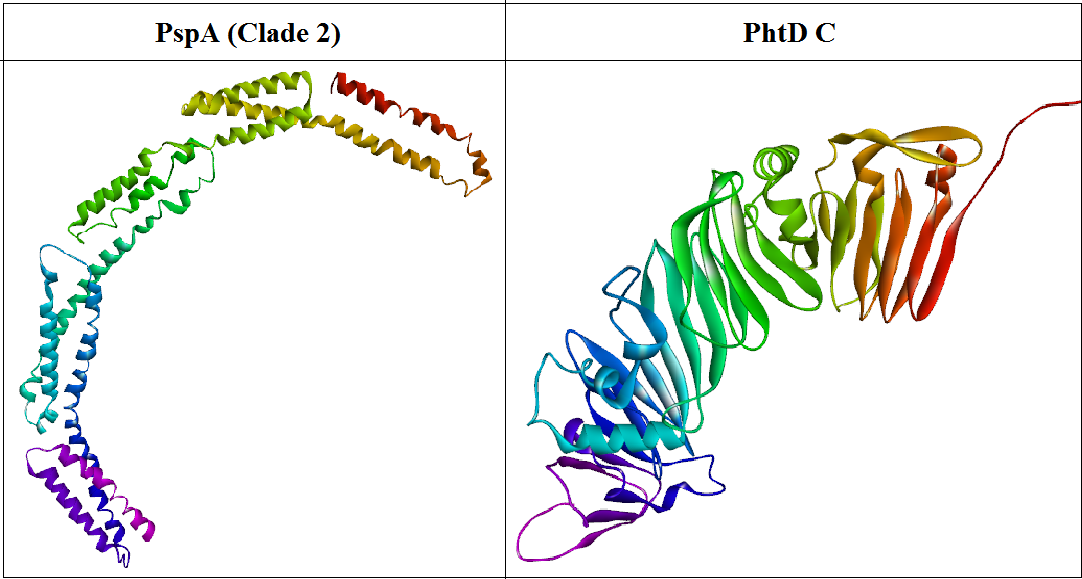


**Supplementary Figure S3. The 3D models of PspA2 and PhtD-C.** The predicted and refined 3D structures were visualized by the Discovery Studio Visualizer.


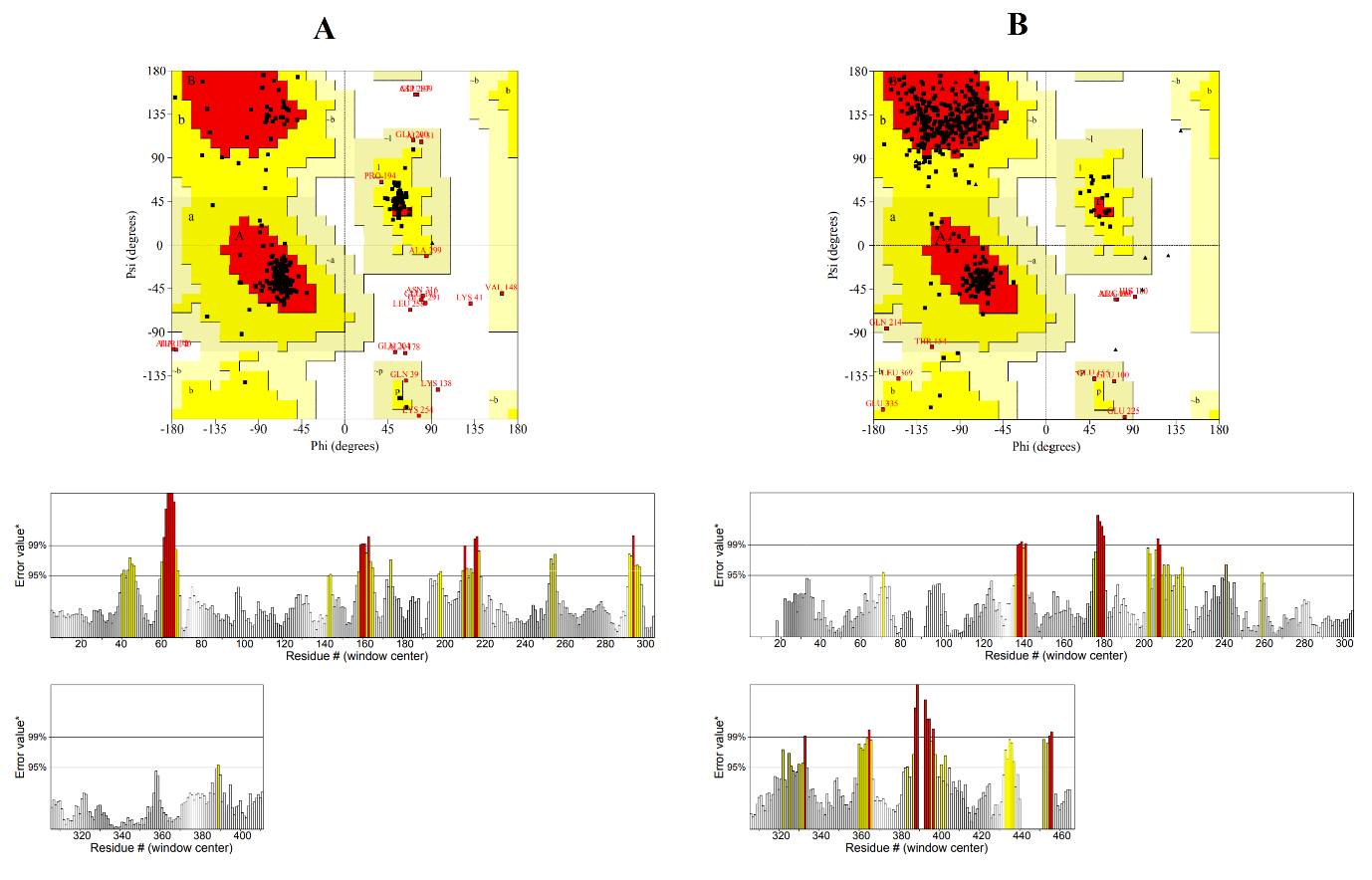


**Supplementary Figure S4. The validation of refined model of PspA2 (A) and PhtD-C (B) with PROCHECK and ERRAT.** Ramachandran plot of the structure of PspA2 or PhtD-C represents 82.1% or 87.2% residues in favored regions, respectively. In the ERRAT plot, the overall quality factor of structure of PspA2 or PhtD-C is 88.69% or 85.23%, respectively.


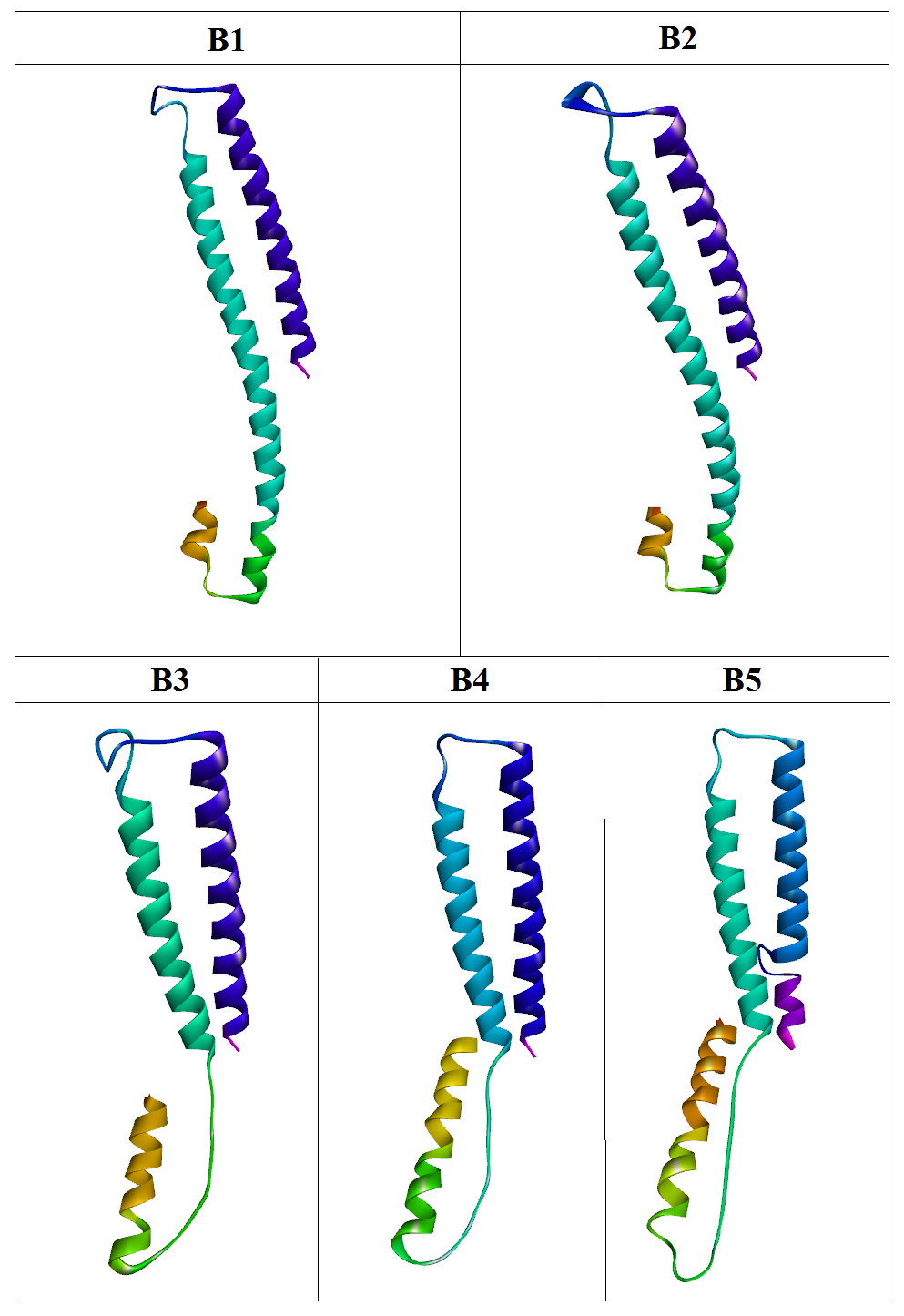


**Supplementary Figure S5. The 3D models of B regions of PspA1-5.** The homology modeled structures of B regions of PspA clades 1 to 5 (B1 to 5, respectively) visualized by Discovery Studio Visualizer.


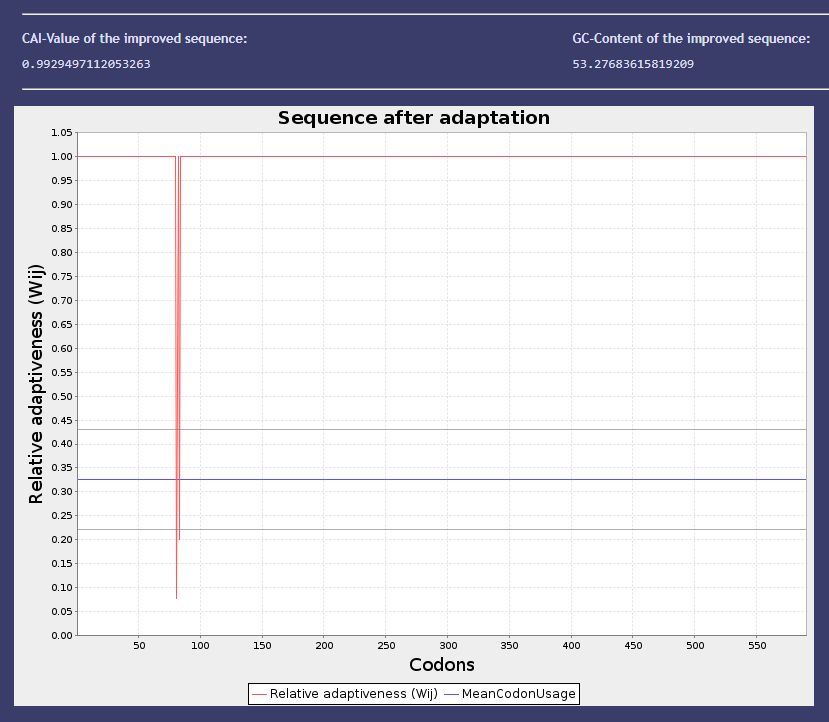


**Supplementary Figure S6. Codon optimization results of the designed construct.** Codon optimization was performed using JCAT and codons were adapted for efficient expression in E.coli K12. The CAI index and GC content of the optimized sequence were 0.99 and 53.27%, respectively.
